# TOR signaling regulates GPCR levels on the plasma membrane and suppresses the *Saccharomyces cerevisiae* mating pathway

**DOI:** 10.1016/j.jbc.2025.110700

**Published:** 2025-09-11

**Authors:** Nicholas R. Leclerc, Toby M. Dunne, Sudati Shrestha, Cory P. Johnson, Joshua B. Kelley

**Affiliations:** 1 Department of Molecular and Biomedical Sciences, University of Maine, Orono, Maine, USA; 2 Graduate School of Biomedical Science and Engineering, University of Maine, Orono, Maine, USA

**Keywords:** GPCR, Ste2, TOR, TORC1, TORC2, *Saccharomyces cerevisiae*, mating response, nitrogen starvation, rapamycin

## Abstract

Target of rapamycin (TOR) complexes and G protein–coupled receptors (GPCRs) are crucial signaling hubs that coordinate adaptive responses to environmental inputs. While GPCR-mediated regulation of TOR has been extensively studied, little is known about TOR-mediated regulation of GPCRs. Here, we establish TOR as a regulator of GPCR signaling *via* its control of receptor endocytosis in the yeast mating system. By pairing fluorescence microscopy with yeast genetic approaches, we identify the machinery that bridges TOR nutrient sensing to internalization of the mating GPCR, Ste2. We found that TORC1 inhibition drives internalization of Ste2 through TORC2, the kinase Ypk1, and the α-arrestins Rod1 and Rog3. Furthermore, we find that Atg8, a central player in autophagy, is employed during mating to deliver active receptor to the vacuole (lysosome), suppressing the mating pathway. These results demonstrate that TOR regulates the localization and signaling of the yeast mating GPCR in both ligand-dependent and ligand-independent contexts. We found that TORC2 activity is required for both rapamycin-driven and pheromone-driven endocytosis of Ste2. These pathways are highly conserved suggesting that TOR regulation of GPCRs may be a broadly conserved mechanism for integrating competing signals involving metabolic state and external communications.

The central metabolic regulator target of rapamycin (TOR) kinase is conserved across eukaryotes (1). TOR kinases were first discovered in yeast as Tor1 and Tor2 (2, 3). Subsequently, the mammalian mTOR was identified with highly conserved mechanisms of action (4, 5, 6, 7). Tor kinases assemble into two distinct protein complexes, TORC1 and TORC2 (mTORC1 and mTORC2 in mammals) (8). These complexes regulate metabolism, growth, autophagy, and endocytosis in response to various stressors (9, 10). While mammalian cells harbor one mTOR protein that assembles into either complex, *Saccharomyces cerevisiae* has two, Tor1 and Tor2 (8). This is likely due to whole genome duplication, a significant evolutionary event that led to the generation of multiple copies of preexisting proteins (11). While Tor1 assembles into TORC1, Tor2 may assemble into both TORC1 and TORC2 (8, 12).

TORC1 resides in the perivacuolar space (13) and is primarily responsive to available nutrients, such as amino acids (14). In nutrient-rich environments, TORC1 is active and phosphorylates downstream effectors to enhance anabolism and protein synthesis while repressing catabolism and the cellular recycling process known as autophagy. Bulk (nonselective) autophagy can be induced pharmacologically by rapamycin (15), a macrolide that forms a complex with Fpr1 (FKBP12 in humans) to directly inhibit TORC1 (2, 5, 6, 7). This results in the formation of double-membraned vesicles, known as autophagosomes, which engulf cytoplasmic cargo as their membranes grow, mature, and close. Autophagosomes ultimately traffic to the vacuole, leading to the destruction of both the vesicle and its internal cargo. This resupplies the cell with free metabolites, prolonging cell survival during nutritional stress (16).

Unlike TORC1, TORC2 is insensitive to rapamycin (8), resides at the cytoplasmic face of the plasma membrane (PM) (17, 18), and responds to PM stressors such as altered membrane tension and sphingolipid depletion (19, 20, 21). TORC2 regulates actin polarization (22, 23, 24, 25, 26) and sphingolipid synthesis (27, 28, 29), resulting in TORC2 having broad control over endocytosis (30, 31). TORC2 is best understood to regulate PM homeostasis and endocytosis through its essential downstream effector Ypk1 (homolog of mammalian SGK1) (29, 30, 32, 33), although it may also do so indirectly through mechanisms that alter PM tension (26, 34). Therefore, TORC2 uses a combination of protein phosphorylation cascades and physical PM mechanics to regulate endocytosis and maintain PM homeostasis in response to membrane stress. Together, TORC1 and TORC2 each serve as unique signaling nexuses that promote survival of cells facing stressful conditions.

The TOR complexes on their own are crucial sensors of environmental stressors (9, 10, 19, 35, 36), a function that is necessary to coordinate adaptive responses against unfavorable conditions. G-protein–coupled receptors (GPCRs) are also critical sensors of extracellular cues (37, 38), many of which organize signaling relays to regulate TOR activity. The regulatory effect of GPCRs, whether positive or negative, varies between systems and cell types (39). For instance, several GPCRs that upregulate PKA activity lead to a reduction in mTORC1 activity, such as the β2-adrenergic receptor and the glucagon receptor (40). Conversely, GPCR activation may also upregulate mTORC1 activity, such as the amino acid receptors T1R1 and T1R3 (41). While there is a growing wealth of knowledge surrounding GPCR-mediated control of the TOR complexes, little is known about how TOR may regulate GPCR signaling.

GPCRs are a conserved family of eukaryotic membrane proteins that serve as sensors of the extracellular environment (37, 38). GPCRs are functionally unique from other cell surface receptors due to their coupling with a heterotrimeric G-protein (consisting of Gα, Gβ, and Gγ subunits) (42). This heterotrimeric G-protein facilitates signal transduction from an active receptor to downstream effectors, including adenylate cyclase (43), phospholipase C (44), PI3K (45), and mitogen-activated protein kinases (46) to induce adaptive responses. In *S. cerevisiae,* mating of haploids is controlled by a GPCR system. These cells secrete pheromones depending on their mating type: MATa cells secrete a-factor and MATα cells secrete α-factor (47, 48, 49). Cells recognize the mating pheromone of the opposite type through their mating GPCRs: Ste2 in MATa (50) and Ste3 in MATα (51). These two GPCRs initiate identical signaling cascades resulting in polarized growth, cell cycle arrest in G1, and transcription of mating genes. Through this GPCR-mediated response, cells are primed to form a mating projection toward the source of pheromone, fuse, and mate with the nearest available partner of the opposite mating type (47, 48, 49).

In the case that multiple cells attempt to mate with the same potential partner, only one can complete the mating process (52). This poses viability and fitness risks for the cells that respond to pheromone but cannot find a mate. Cells that respond to pheromone for prolonged periods of time are at a greater risk of death (53). Moreover, cells that continue to try and mate will spend more time, energy, and nutrients forming an obsolete mating projection, giving up the potential to produce more progeny and maintain their biological fitness. Therefore, cells must constrain the activation of their pheromone receptors. Cells have programmed mechanisms to downregulate GPCR signaling following pheromone recognition. This occurs primarily through the 1) inactivation of the G-protein (54, 55) and 2) internalization of the GPCR (56). These processes desensitize the cell to pheromone at the ligand, receptor, and G-protein level, downregulating the mating pathway and preventing further pheromone signaling.

Endocytosis of the yeast pheromone receptor Ste2 following its activation by pheromone is well understood. The GPCR activation results in C-terminal phosphorylation by yeast casein kinases (Yck1 and Yck2) (57, 58, 59). This phosphorylation allows for the recruitment of α-arrestins (Rod1 and Rog3) (56, 60, 61). The receptor tail is then multimonoubiquitinated (62), allowing for recruitment of epsin-like proteins (Ent1 and Ent2) that promote assembly of endocytic machinery (63, 64) and drive internalization of the receptor (65, 66). Following receptor-mediated endocytosis, Ste2 is trafficked to the vacuole (lysosome) for degradation (67, 68), whereas Ste3 localizes at the endosome and is recycled (69). Both scenarios lead to the desensitization of the mating receptors to pheromone (56, 60).

Pheromone-induced internalization of Ste2 and downregulation of the mating pathway is well understood. However, it has been reported that various environmental stresses can regulate the mating pathway. Hyperosmotic stress dampens pheromone-induced MAPK activation and gene expression (70). Furthermore, glucose starvation represses G-protein signaling (71) and promotes Ste2 internalization (72). These instances highlight how cells prioritize responding to stress signals over mating. Interestingly, mass spectrometry–based proteomics reports nitrogen starvation to decrease overall Ste2 levels in the cell (73). However, when glucose starvation was shown to promote Ste2 internalization, TORC1 activity was maintained, and autophagy was inhibited. Thus, there are gaps in the understanding of how nitrogen starvation specifically regulates Ste2.

During the pheromone response, cytoplasmic proteins have been found to accumulate in the vacuole (74), a hallmark of autophagy despite the lack of nutritional stress. As mentioned above, proteomic approaches indicate that nitrogen starvation diminishes Ste2 levels (73). These data led us to hypothesize that TOR inhibition and autophagy machinery may serve to drive Ste2 endocytosis and suppression of the pheromone signaling pathway. Here, we show that TOR signaling regulates the surface presentation of both mating GPCRs in *S. cerevisiae* during TORC1 inhibition, leading to the internalization of the mating receptors. Coincident with this altered receptor localization, we find that TORC1 inhibition dampens pheromone signaling and the ability to mate. Furthermore, we identify key signaling components linking TORC1 to Ste2 internalization, such as TORC2 and its effector Ypk1. We then assess whether TORC effectors are utilized to suppress the mating response following exposure to pheromone. We find that Ypk1 significantly represses the mating pathway. We also show that the central autophagy protein Atg8 aids in the vacuolar targeting of Ste2 to the vacuole, suppressing the mating pathway. Thus, TOR complexes and their effectors can regulate GPCRs in both ligand-dependent and ligand-independent contexts.

## Results

### Nutrient availability regulates surface presentation of the mating GPCR Ste2

**Figure 1:**
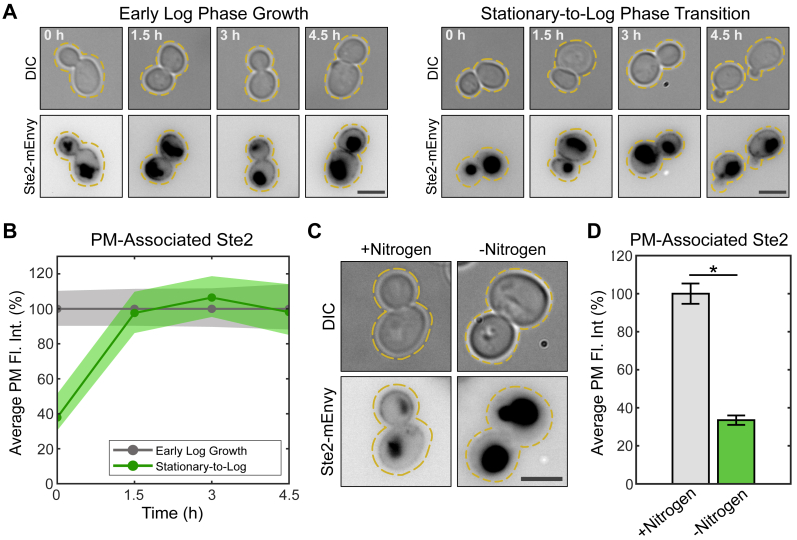
**Nutrient availability regulates Ste2 levels at the PM.***A*, representative images of cells expressing Ste2-mEnvy maintained in the log phase of growth (*left panels*) (n > 130 cells) or grown to stationary phase and then diluted in replete media (*right panels*) (n > 90 cells) for 4.5 h. Scale bars represent 4 μm. *B*, quantification of Ste2-mEnvy mean fluorescent intensity at the plasma membrane every 1.5 h either during early log phase growth or growth from stationary phase into replete media. Measurements were normalized to the mean of “early log growth” values at each time point to produce percentages. *Shaded areas* represent 95% bootstrap confidence intervals. *C*, representative images of cells expressing Ste2-mEnvy grown in SCD media (+Nitrogen) (n = 252 cells) or low nitrogen SCD media (-Nitrogen) (n = 568 cells) for 4.5 h. The scale bar represents 4 μm. *D*, quantification of Ste2-mEnvy mean fluorescent intensity at the plasma membrane in cells grown in SCD media (+Nitrogen) or low nitrogen SCD media (-Nitrogen) for 6 h (n > 250 cells). Measurements were normalized to the mean of the +Nitrogen group to produce percentages. Error bars represent ±SEM. ∗ = *p* < 0.001 by two-sample two-tailed unequal variance *t* test. mEnvy, monomeric Envy; PM, plasma membrane; SCD, Synthetic Complete plus Dextrose.

Glucose starvation has been reported to drive Ste2 internalization, and nitrogen starvation has been reported to reduce Ste2 levels (72, 73). Furthermore, TORC1 can upregulate or downregulate endocytosis in different contexts (72, 73, 75, 76, 77). Nitrogen starvation impacts on Ste2 have not been investigated beyond the proteomics screen that found reduced Ste2 levels (73). Therefore, we first set out to validate the effects of nitrogen starvation signaling on Ste2 internalization. To test this, we examined cells expressing Ste2-monomeric Envy (mEnvy) from a culture grown to saturation. Cells were either grown to the stationary phase of growth (A_600_ > ∼1.2–1.5) to create a nutrient-deprived environment or maintained in the nutrient-rich mid-log phase of growth (A_600_ < 0.8) and then imaged by fluorescence microscopy (Fig. 1*A*). Cells in the mid-log phase showed typical receptor localization patterns, Ste2 was present on the PM and inside the vacuole of the cell. However, cells grown to stationary phase showed a 62% reduction in abundance at the PM (Fig. 1*B*). We tested whether this was a reversible effect by diluting stationary phase cells and supplementing them with fresh nutrients over 4.5 h. The control group was sustained in mid-log growth by regular media changes. Previously-starved cells showed a complete recovery of PM-associated Ste2 after 1.5 h in replete media (Fig. 1*B*). This indicates that the available nutrients determine the spatial localization of the mating receptor.

While saturated cultures should be depleted of both nitrogen and glucose, we were specifically interested in TOR signaling and its regulation by nitrogen depletion (14). Cells were kept at a subsaturating density (A_600_ = 0.2–0.8) in standard Synthetic Complete plus Dextrose (SCD) media and then diluted into low-nitrogen SCD media for 4.5 h. Cells were constantly maintained in the mid-log phase of growth throughout the experiment and imaged by fluorescence microscopy (Fig. 1*C*). Cells in nitrogen-limited media showed a 66% reduction of Ste2 abundance at the cell periphery while in the mid-log phase of growth (Fig. 1*D*). Thus, localization of Ste2 to the PM is specifically sensitive to nitrogen levels in the media.

### TORC1 alters Ste2 PM levels

**Figure 2:**
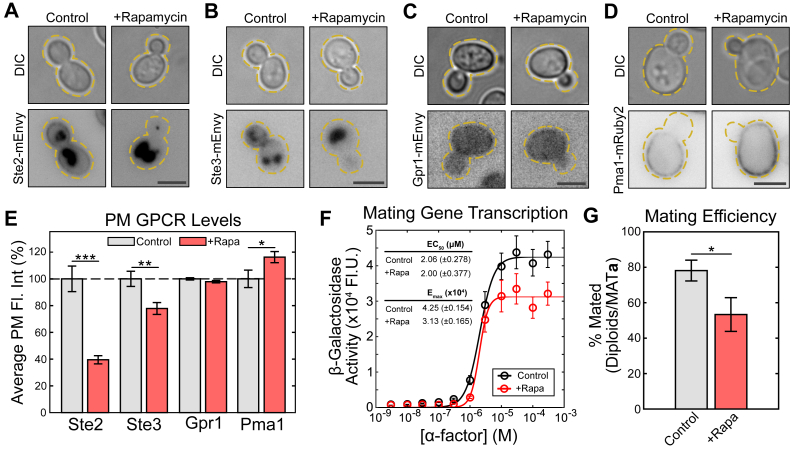
**Inhibition of TORC1 reduces mating GPCR levels at the PM and represses the mating pathway.***A*-*D*, representative images of cells expressing (*A*) Ste2-mEnvy, (*B*) Ste3-mEnvy, (*C*) Gpr1-mEnvy, and (*D*) Pma1-mRuby2 treated with or without 0.2 μM rapamycin for 2 h. Scale bars represent 4 μm. *E*, quantification of Ste2-mEnvy, Ste3-mEnvy, Gpr1-mEnvy, and Pma1-mRuby2 mean fluorescent intensities at the plasma membrane (-Rap: Ste2 n = 212 cells, Ste3 n = 285 cells, Gpr1 n = 197 cells, Pma1 n = 270 cells. +Rap: Ste2 n = 277 cells, Ste3 n = , Gpr1 n = 214 cells, Pma1 n = ). Measurements were normalized to the mean of the untreated groups to produce percentages. Error bars represent ±SEM. ∗ = *p* < 0.05, ∗∗ = *p* < 0.01, and ∗∗∗ = *p* < 0.001. *F*, β-galactosidase (pFUS1-LacZ) mating transcription assays of cell cultures pretreated with or without 0.2 μM rapamycin for 2 h (n > 10 colonies). Error bars represent ±SEM. EC_50_ and E_max_ are reported with ±95% confidence intervals. *G*, quantitative mating assays in cells pretreated with or without 0.2 μM rapamycin for 2 h (n = 5 assays). Error bars represent ±SEM. ∗ = *p* < 0.05 by two-sample one-tailed unequal variance *t* test. GPCR, G-protein–coupled receptor; mEnvy, monomeric Envy; PM, plasma membrane; TORC, target of rapamycin complex.

Having confirmed that nitrogen availability controls Ste2 levels on the PM, we hypothesize that the nitrogen-sensitive TORC1 may control the cell signaling that leads to changes in receptor localization. Rapamycin specifically represses TORC1 activity, mimicking the effects of nitrogen depletion (2, 5, 6, 7). We treated cells with 0.2 μM rapamycin in SCD media during mid-log growth for 2 h, followed by imaging cells expressing Ste2-mEnvy using fluorescence microscopy. (Fig. 2*A*). Rapamycin treatment led to a 60% reduction in peripheral Ste2 levels (Fig. 2*E*), showing TORC1 signaling is responsible for starvation-induced internalization of the receptor. These results indicate that TORC1 inhibition promotes Ste2 internalization.

There are three GPCRs in yeast: the mating receptors Ste2 and Ste3, and the sugar receptor Gpr1 (78). To test if TORC1 inhibition impacts other GPCRs, or whether it is selective for Ste2, we generated strains with Ste3 and Gpr1 tagged with mEnvy. We treated these cells with 0.2 μM rapamycin for 2 h and then observed their PM levels through fluorescence microscopy (Fig. 2, *B*, *C* and S1). Ste3 levels at the PM decreased by 22%, while Gpr1 abundance remained unchanged (Fig. 2*E*). To test if other PM proteins were internalized, we labeled the proton pump Pma1 (79) with mRuby2 and performed the same rapamycin experiment as above (Fig. 2*D*). Pma1 localization to the PM showed a 16% increase in the presence of rapamycin (Fig. 2*E*). These results show that TORC1 inhibition does not cause indiscriminate endocytosis of integral membrane proteins, as demonstrated by the increase in the abundance of Pma1 and unaltered levels of Gpr1 at the periphery. Further, this TORC1-dependent process is selective among GPCRs, as it specifically downregulates both mating GPCRs Ste2 and Ste3, while not impacting Gpr1.

The reduced surface presentation of Ste2 during TORC1 inhibition led us to hypothesize that rapamycin-treated cells would have a diminished pheromone response. To test this, we measured pheromone-dependent transcription in the presence and absence of rapamycin using a pheromone-responsive β-galactosidase assay (Fig. 2*F*). In this assay, we use a plasmid with β-galactosidase driven by the pheromone-responsive *FUS1* promoter (pFUS-LacZ) (80). Therefore, we can measure the dose-response of pheromone-induced transcription based on the activity of the β-galactosidase reporter. We found that rapamycin treatment reduces the maximal transcriptional output (E_max_) of mating genes by 26%. Rapamycin did not affect the EC_50_. Thus, TORC1 inhibition both removes Ste2 from the membrane and suppresses Ste2-induced transcription.

### TORC1 inhibition suppresses mating

Loss of receptor from the PM led us to hypothesize that mating would be less likely to happen when TORC1 activity is inhibited. To test this, we performed quantitative mating assays (81) in cells treated with or without rapamycin (Fig. 2*G*). Under control conditions, 78% of untreated cells can successfully mate over 5 h, consistent with previously reported levels (82). In contrast, only 53% of cells treated with rapamycin successfully mated. Thus TORC1 activity normally promotes efficient mating, while decreased signaling consistent with nitrogen stress makes successful mating less likely.

### TORC1-dependent endocytosis of Ste2 occurs through clathrin-mediated endocytosis and TORC2 signaling

**Figure 3:**
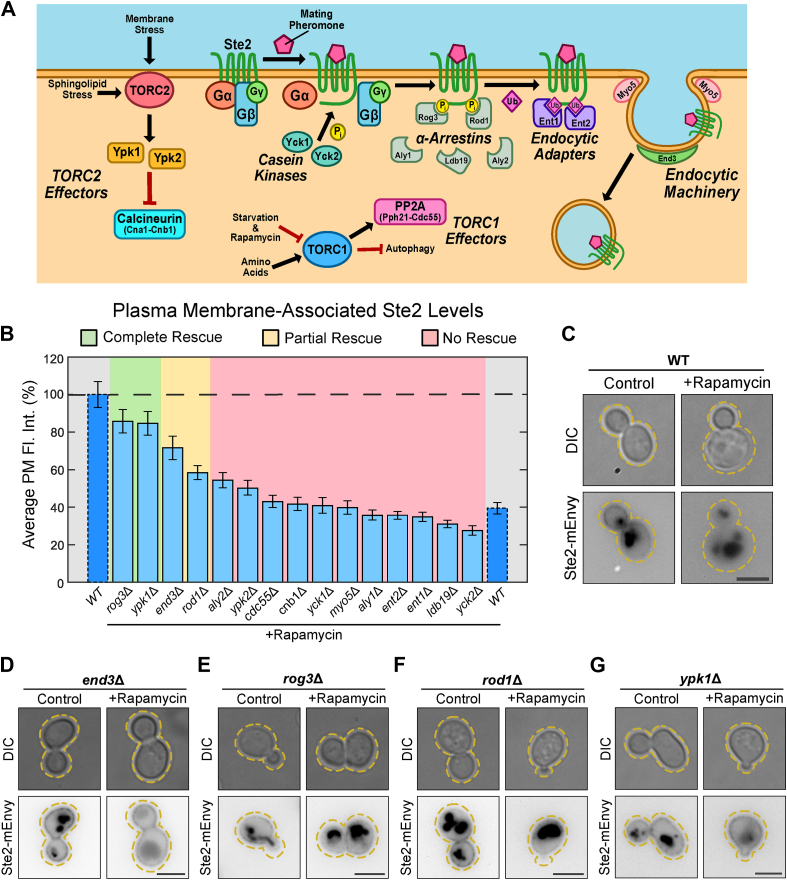
**TORC1 inhibition leads to CME of Ste2 through α-arrestins and Ypk1.***A*, to identify candidates involved in TORC1-mediated endocytosis, we deleted multiple genes that are involved in TORC signaling, receptor endocytosis, and CME. Casein kinases (57, 58, 59), α-arrestins (56, 60, 61, 127), and epsin-like proteins (65, 66) are all involved in priming the pheromone receptors for CME during mating. CME utilizes motors (128) and cytoskeletal organizers (83, 84) to form then endocytic pit that matures into an endosome. During starvation, TORC1 activity is reduced, downregulating PP2A activity (129). TORC2 activates Ypk1 and Ypk2 (29, 36, 130), which repress major cell regulators such as calcineurin (32). *B*, quantification of mean Ste2-mEnvy on the plasma membrane in the indicated strain, with or without 0.2 μM rapamycin for 2 h (n > 170 cells). Measurements were normalized to the mean of the untreated groups (Fig. S1) to produce percentages. One-way ANOVA was used to analyze variance between treated and untreated mutant and WT cells. Tukey’s HSD test was used as a multiple comparison test to compare means between groups. Based on ANOVA results, genetic mutants treated with rapamycin were grouped into one of three categories: no rescue (*red*), partial rescue (*yellow*), and complete rescue (*green*). Mutants in the no rescue category were only significantly different (*p* < 0.05) from untreated WT cells. Mutants in the partial rescue category were significantly different from both treated and untreated WT cells. Mutants in the complete rescue category were only significantly different from rapamycin-treated WT cells. Error bars represent ±SEM. Bars with *dashed outlines* show data reported in Figure 2*E*. *C*–*G*, representative images of (*C*) WT, (*D*) *end*3Δ, (*E*) *rog*Δ, (*F*) *rod*1Δ, and (*G*) *ypk*1Δ cells expressing Ste2-mEnvy treated with or without 0.2 μM rapamycin for 2 h. Scale bars represent 4 μm. CME, clathrin-mediated endocytosis; HSD, honestly significant difference; mEnvy, monomeric Envy; TORC, target of rapamycin complex.

We set out to determine the components in the pathway linking TORC1 inhibition to loss of Ste2 from the PM. To accomplish this, we constructed 15 strains with deletions of genes known to be involved in TORC signaling, Ste2 endocytosis, and clathrin-mediated endocytosis (CME) (Fig. 3*A*). These cells were treated with 0.2 μM rapamycin for 2 h, and peripheral Ste2 levels were measured using fluorescence microscopy. Each deletion mutant was then grouped into one of three categories based on whether they were statistically equivalent (by ANOVA followed by multiple comparison) to the control, the rapamycin treatment, or neither when treated with rapamycin. The three groups are as follows: 1) No rescue (impact of rapamycin on receptor localization is not changed from WT); 2) Complete rescue (Fig. 3*B*) (addition of rapamycin does not impact Ste2 localization in mutant) and; 3) Partial rescue (rapamycin effect on receptor is statistically different from WT cells with or without rapamycin). Many of the untreated deletion mutants show altered receptor abundance from untreated WT cells (Figs. S1 and S2), adding another layer of complexity to the function of these proteins in maintaining typical receptor levels. However, we are specifically focused on comparing the impact of rapamycin on receptor localization within a strain and how that compares to the impact of rapamycin on WT cells.

Multiple proteins involved in CME of Ste2 were found to be involved in rapamycin-induced endocytosis of Ste2. We found that deleting *END3* partially blocks the effect of rapamycin on Ste2 localization on the PM (Fig. 3, *B* and *D*). End3 is an adaptor protein that facilitates the internalization step in CME. Cells that lack End3 show defects in endocytosis and have been reported to reduce internalization of Ste2 by ∼60 to 80% (83, 84). CME in these cells is defective, but not entirely absent. We also saw that deletion of the α-arrestin Rod1 partially blocked the effect of rapamycin on Ste2 localization (Fig. 3, *B* and *F*). In contrast, deleting the α-arrestin Rog3 completely blocked rapamycin-induced internalization of Ste2 (Fig. 3, *B* and *E*). The α-arrestins Rod1 and Rog3 are paralogs that facilitate CME and desensitization of active Ste2 in response to pheromone (56, 60). To our surprise, Ste2 levels were both reduced in untreated *rod1*Δ and *rog3*Δ cells compared to untreated WT cells (Fig. S1). However, both visual inspection and comparison to *ldb19*Δ +rapamycin indicate that Ste2 levels can go lower, and thus these strains are still within the dynamic range of the assay. The ability of *ROD1*, *ROG3*, or *END3* deletion to block rapamycin effects indicates that CME plays a role in the internalization of Ste2 in response to TORC1 inhibition.

**Figure 4:**
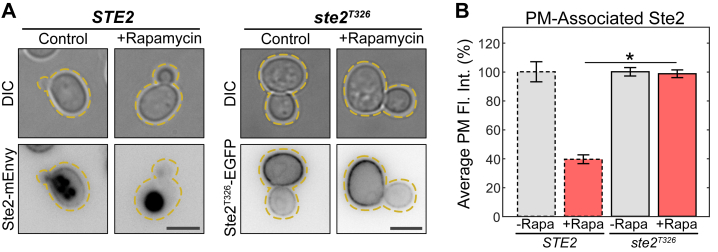
**The C terminus of Ste2 is required for TORC1-mediated endocytosis.***A*, representative images of cells expressing Ste2-mEnvy and Ste2^T326^-EGFP treated with (n = 184 cells) and without (n = 132 cells) 0.2 μM rapamycin for 2 h. Scale bar represents 4 μm. *B*, quantification of Ste2-mEnvy and Ste2^T326^-EGFP mean fluorescent intensities at the plasma membrane. Measurements were normalized to the mean of the untreated groups to produce percentages. One-way ANOVA paired with Tukey’s HSD test was used to compare means between groups. Error bars represent ±SEM. ∗ = *p* < 0.001. Bars with *dashed outlines* show data reported in Figure 2*E*. HSD, honestly significant difference; mEnvy, monomeric Envy; TORC, target of rapamycin complex.

Rod1 and Rog3 are known to associate with the phosphorylated C terminus of active Ste2 (56, 60), suggesting that C-terminal modification of Ste2 is required for this internalization event. To test this, we replaced the C terminus of Ste2 with EGFP (*ste2*^*T326*^*-EGFP*) in cells and then treated with rapamycin as described above (Fig. 4*A*). Ste2^T326^ levels at the PM did not change when treated with rapamycin (Figs. 4*B* and S1). This confirms that the internalization of the receptor is reliant on the C-terminal tail, which has a well-characterized role in CME of the receptor (62, 85, 86, 87). Finally, we found that deleting *YPK1* completely blocks the effect of rapamycin on Ste2 PM levels (Fig. 3, *B* and *G*). Interestingly, *ypk1*Δ also showed a lower level of Ste2 on the PM in the control condition (Fig. S1). However, *ypk1*Δ cells do not show a complete loss of receptor, as multiple other strains are able to achieve lower receptor levels (Fig. S1).

**Figure 5:**
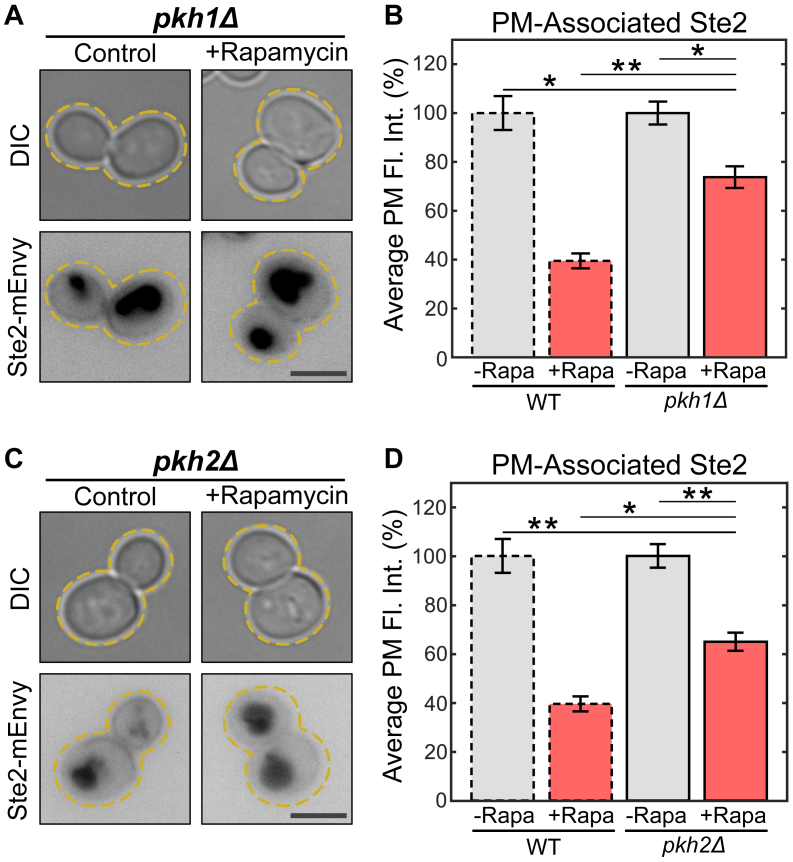
**The Ypk1 activators Pkh1 and Pkh2 contribute to TORC1-mediated Ste2 endocytosis.***A*, representative images of *pkh1Δ* cells expressing Ste2-mEnvy treated with (n = 186 cells) or without (n = 195 cells) 0.2 μM rapamycin for 2 h. *B*, quantification of Ste2-mEnvy mean fluorescent intensities at the plasma membrane. Measurements were normalized to the mean of the untreated groups to produce percentages. One-way ANOVA paired with Tukey’s HSD test was used to compare means between groups. *C*, representative images of *pkh2Δ* cells expressing Ste2-mEnvy treated with (n = 135 cells) or without (n = 162 cells) 0.2 μM rapamycin for 2 h. *D*, quantification of Ste2-mEnvy mean fluorescent intensities at the plasma membrane. Measurements were normalized to the mean of the untreated groups to produce percentages. One-way ANOVA paired with Tukey’s HSD test was used to compare means between groups. All error bars represent ±SEM. ∗ = *p* < 0.01, ∗∗ = *p* < 0.001. All bars with *dashed outlines* show data reported in Figure 2*E*. Scale bars represent 4 μm. mEnvy, monomeric Envy; HSD, honestly significant difference.

Ypk1 and its paralog Ypk2 are kinases that act downstream of TORC2, carrying out all of the complex’s essential functions (22, 29, 33). These proteins are active when they are phosphorylated by kinases Pkh1 and Pkh2 (88, 89, 90). Therefore, we deleted *PKH1* and *PKH2* in cells expressing Ste2-mEnvy and treated them with rapamycin to see if upstream components of Ypk1 also rescue Ste2 abundance at the PM (Fig. 5, *A* and *C*). We found that both deletions partially rescued Ste2 abundance at the PM (Figs. 5, *B*, *D* and S1). Pkh1 and Pkh2 are functionally redundant (88), therefore it is unsurprising that the rescue seen in either strain is incomplete. While Pkh1 and Pkh2 may not be restricted to signaling solely through Ypk1, the concomitant rescue effects on the receptor from both the Pkh proteins and Ypk1 suggest a role in Ste2 internalization during TORC1 inhibition, most likely through partially redundant Ypk1 activation.

**Figure 6:**
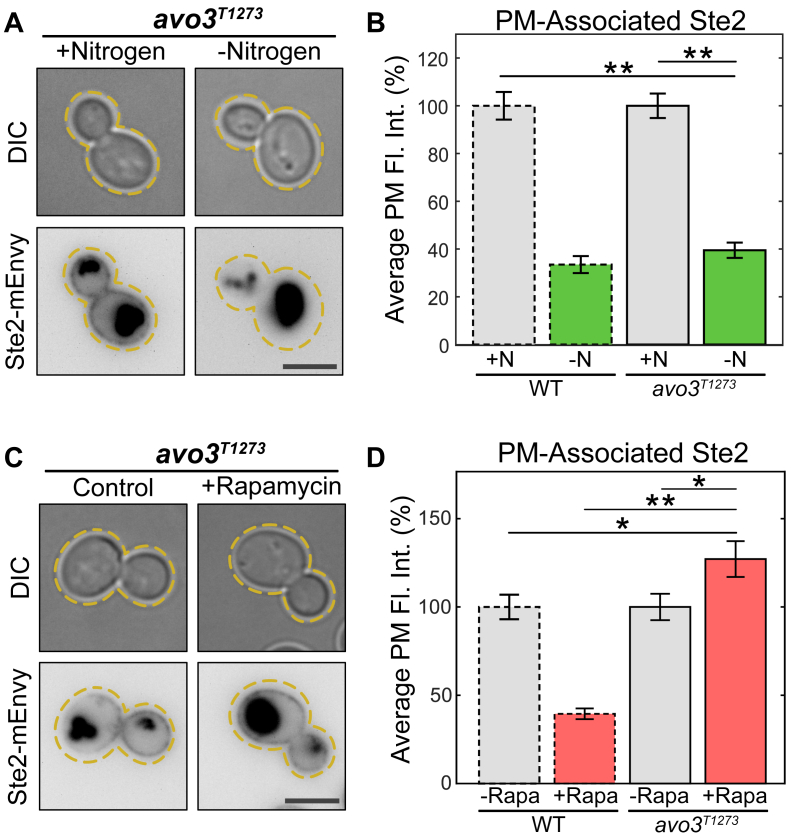
**TORC2 activity directs TORC1-mediated Ste2 endocytosis.***A*, representative images of cells expressing Ste2-mEnvy and avo3^T1273^ treated with (n = 233 cells) and without (n = 139 cells) 0.2 μM rapamycin for 2 h. *B*, quantification of Ste2-mEnvy mean fluorescent intensities at the plasma membrane. Measurements were normalized to the mean of the untreated groups to produce percentages. One-way ANOVA paired with Tukey’s HSD test was used to compare means between groups. *C*, representative images of cells expressing Ste2-mEnvy and avo3^T1273^ grown in SCD media (+N) (n = 141 cells) or low nitrogen SCD media (-N) (n = 109 cells) for 6 h. *D*, quantification of Ste2-mEnvy mean fluorescent intensities at the plasma membrane. Measurements were normalized to the mean of the untreated groups to produce percentages. One-way ANOVA paired with Tukey’s HSD test was used to compare means between groups. All error bars represent ±SEM. ∗ = *p* < 0.05, ∗∗ = *p* < 0.001. All bars with *dashed outlines* show data reported in Figure 2*E*. Scale bars represent 4 μm. HSD, honestly significant difference; mEnvy, monomeric Envy; SCD, Synthetic Complete plus Dextrose; TORC, target of rapamycin complex.

Ypk1 is upregulated by TORC2 in response to stress (19, 22, 33, 90). Next, we tested whether TORC2 signaling was required for Ste2 internalization in response to TORC1 inhibition. The core protein of TORC2, Tor2, is essential, and TORC2 is not normally sensitive to rapamycin (8). Therefore, we could not delete *TOR2* or inhibit it as in our other experiments. Instead, we made TORC2 sensitive to rapamycin by truncating the TORC2 subunit Avo3. Avo3 is a scaffold protein that helps maintain the structural integrity of TORC2. The C terminus of Avo3 masks the rapamycin-binding site residing on Tor2, rendering the complex insensitive to rapamycin treatment. As such, deleting the C terminus of Avo3 confers rapamycin sensitivity to TORC2 (91, 92).We reasoned that if TORC2 is responsible for Ste2 internalization upon inhibition of TORC1, and then nitrogen starvation would still drive Ste2 internalization in the Avo3 truncation mutant, as TORC2 function should be maintained. However, rapamycin treatment would now inhibit signaling through both TORC1 and TORC2, and Ste2 would remain on the PM during rapamycin treatment. We performed both nitrogen starvation and rapamycin treatment as above. As expected, nitrogen starvation was still able to drive Ste2 internalization in the *avo3*^*T1273*^ strain (Fig. 6, *A* and *B*). However, when *avo3*^*T1273*^ cells were treated with rapamycin, Ste2 did not display normal rapamycin-induced internalization (Figs. 6, *C*, *D* and S1). These results together confirm that inhibition of TORC1 promotes Ste2 internalization through TORC2 signaling.

### Rapamycin and pheromone do not show a synergistic effect on Ste2 internalization

**Figure 7:**
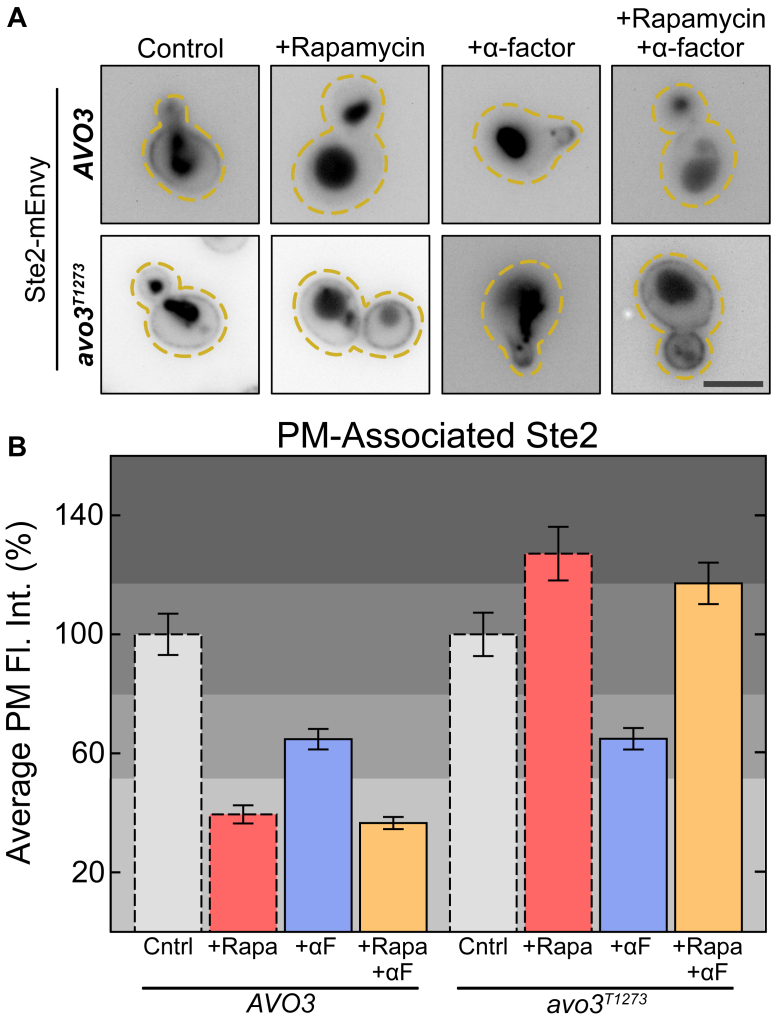
**TORC2 is required for pheromone-induced endocytosis.***A*, representative images of *AVO3* and *avo3*^*T1273*^ cells expressing Ste2-mEnvy treated with either 0.2 μM rapamycin (*AVO3* n = 277 cells; *avo3*^*T1273*^ n = 233 cells), 10 μM α-factor (*AVO3* n = 178 cells; *avo3*^*T1273*^ n = 160 cells), both (*AVO3* n = 306 cells; *avo3*^*T1273*^ n = 258 cells), or neither (*AVO3* n = 212 cells; *avo3*^*T1273*^ n = 139 cells) for 2 h. The scale bar represents 4 μm. *B*, quantification of Ste2-mEnvy mean fluorescent intensities at the plasma membrane. Measurements were normalized to the mean of the untreated groups to produce percentages. One-way ANOVA paired with Tukey’s HSD test was used to compare means between groups. Rather than provide a full matrix of statistical results per this analysis, the chart has been divided into separate *shaded sections*, where the means of any bars that lie in the same section are *not* significantly different from each other. The mean of *avo3*^*T1273*^ +Rapa +αF lies on the border of two *shaded sections*. This shows that while means within these two sections are significantly different from each other (*e.g., avo3*^*T1273*^ Ctrl and *avo3*^*T1273*^ +Rapa) they are *not* significantly different from *avo3*^*T1273*^ +Rapa +αF. Bars with *dashed outlines* show data reported in either Figure 2*E* (*AVO3*) or Figure 6*D* (*avo3*^*T1273*^). mEnvy, monomeric Envy; TORC, target of rapamycin complex.

We next asked whether there was a synergistic effect between rapamycin-induced endocytosis and pheromone-induced endocytosis, as a synergistic effect would suggest independent regulation of internalization. We treated either WT or *avo3*^*T1273*^ cells expressing Ste2-mEnvy with either α-factor (10 μM) or α-factor (10 μM) and rapamycin (0.2 μM) together. After 2 h of treatment, we imaged Ste2-mEnvy (Fig. 7*A*). In the WT background, addition of rapamycin alone yielded the same levels of receptor on the membrane as rapamycin and α-factor, both of which were lower than α-factor alone (Fig. 7*B*). When TORC2 is inhibited in the *avo3*^*T1273*^ strain, endocytosis of Ste2 is completely abrogated in both rapamycin alone and in the α-factor and rapamycin treatment. In the absence of rapamycin, the *avo3*^*T1273*^ mutation did not affect α-factor–induced receptor endocytosis. We do not see a synergistic effect between addition of pheromone and rapamycin, suggesting shared components.

### Ypk1 suppresses mating transcription in response to pheromone

**Figure 8:**
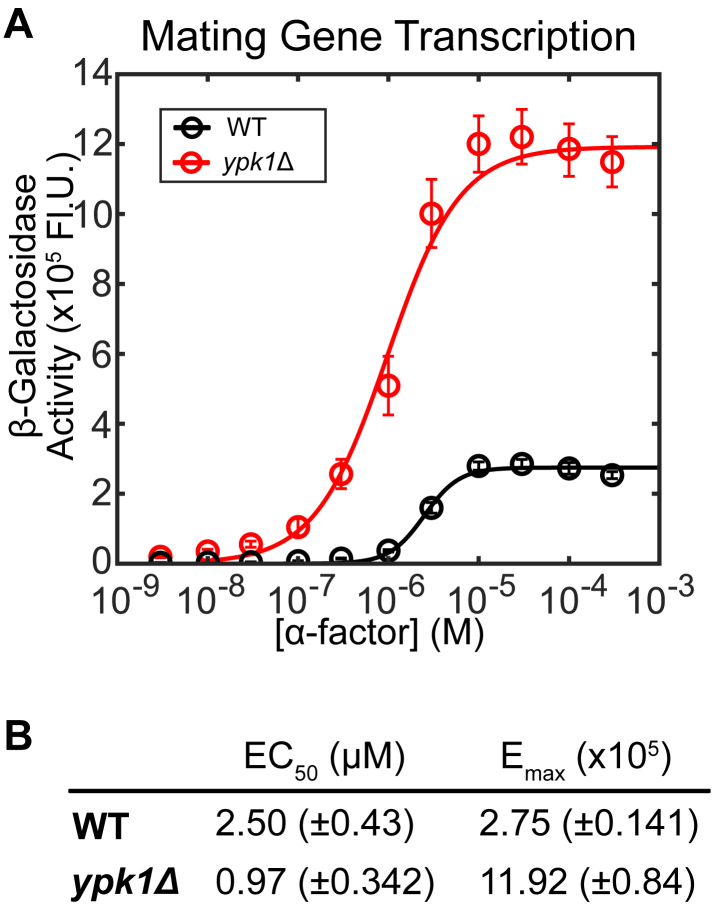
**Ypk1 dampens mating pathway output.***A*, β-galactosidase (pFUS1-LacZ) mating transcription assays of WT (n = 9 colonies) and *ypk1*Δ (n = 9 colonies) cultures. Error bars represent ±SEM. *B*, table of EC_50_ and E_max_ responses to α-factor. EC_50_ and E_max_ are reported with ±95% confidence intervals.

We have found that Ypk1 is required for rapamycin-induced Ste2 endocytosis. We hypothesize that Ypk1 may be responsible for a concomitant reduction in mating transcription during the pheromone response. To test this, we performed a β-galactosidase assay as described above to measure the transcriptional output of cells lacking Ypk1 (Fig. 8*B*). These cells show a remarkable 433% increase in transcription of mating genes and a 39% decrease in EC_50_. This indicates that Ypk1 is a negative regulator of the pheromone pathway in the absence of rapamycin or nutritional stress.

### The autophagy protein Atg8 promotes rapamycin-induced reduction of Ste2 on the PM

**Figure 9:**
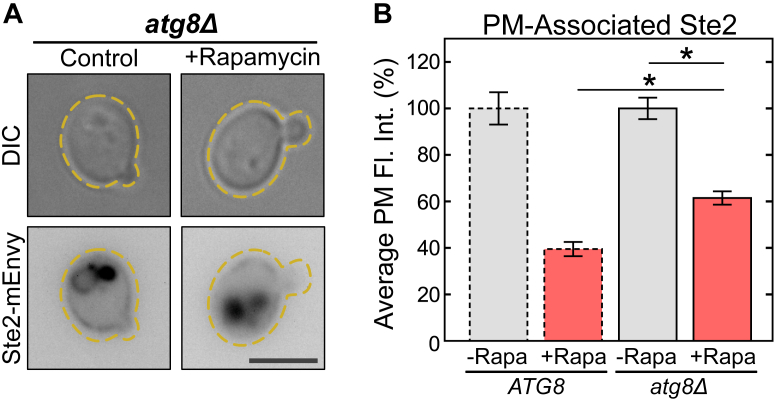
**Atg8 contributes to rapamycin-induced Ste2 internalization.***A*, representative images of *atg8Δ* cells expressing Ste2-mEnvy treated with (n = 303 cells) and without (n = 211 cells) 0.2 μM rapamycin for 2 h. The scale bar represents 4 μm. *B*, quantification of Ste2-mEnvy and mean fluorescent intensities at the plasma membrane. Measurements were normalized to the mean of the untreated groups to produce percentages. One-way ANOVA paired with Tukey’s HSD test was used to compare means between groups. Error bars represent ±SEM. ∗ = *p* < 0.001. Bars with *dashed outlines* show data reported in Figure 2*E*. HSD, honestly significant difference; mEnvy, monomeric Envy.

Atg8 is central to the extension of the autophagosome (93), the double-membrane organelle responsible for engulfment of cellular contents for destruction in the vacuole during autophagy (16, 94). Since our experimental conditions should be activating autophagy, we wondered whether it would impact Ste2 levels on the PM. We generated an *ATG8* deletion strain (*atg8Δ*) and tested the impact of rapamycin on Ste2 localization (Fig. 9). Interestingly, lack of Atg8 leads to a statistically significant increase in Ste2 levels on the PM. Thus, Atg8 promotes the reduction in Ste2 on the PM. Our understanding of Atg8 does not implicate it in endocytosis itself (95, 96, 97), so it seems likely the impact of Atg8 on Ste2 is through endomembrane trafficking.

### Pheromone-induced autophagy represses mating transcription

**Figure 10:**
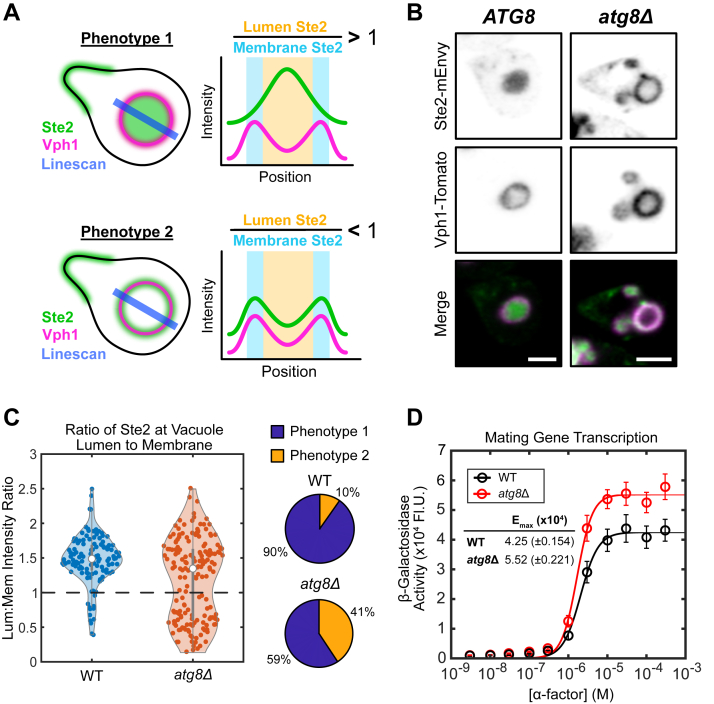
**Pheromone-induced autophagy aids in vacuolar targeting of Ste2 and dampens the mating pathway.***A*, scheme representing the analysis of Ste2-mEnvy at the vacuolar lumen (Vph1-Tomato) and membrane. We have found that mating cells show two primary phenotypes regarding Ste2 localization to the vacuole. Phenotype 1 shows a cell with a vacuole filled with Ste2-mEnvy, whereas phenotype 2 shows an empty vacuole, with more receptor at the vacuolar periphery. Line scans were measured through the center of the largest vacuolar body. The mean fluorescent intensity of Ste2-mEnvy was recorded at the peaks of Vph1-Tomato florescence, thereby reporting Ste2 levels at the vacuolar membrane. The mean fluorescent intensity of Ste2-mEnvy between these peaks was reported as Ste2 levels in the vacuolar lumen. The lumen:membrane ratio of Ste2 would then represent the phenotype each cell presents. Cells with Lum:Mem > 1 presented as phenotype 1 (*full*), whereas cells with a ratio < 1 presented at phenotype 2 (*empty*). *B*, representative images of *ATG8* and *atg8Δ* cells expressing Ste2-mEnvy and Vph1-Tomato treated with 10 μM α-factor. The scale bar represents 2 μm. *C*, violin plots and pie charts representing the distribution of WT (n = 123 cells) and *atg8Δ* (n = 172 cells) cells presenting either phenotype 1 or phenotype 2 when treated with 10 μM α-factor. *D*, β-galactosidase (pFUS1-LacZ) mating transcription assays of WT (n = 15 colonies) and *atg8*Δ cultures (n = 9 colonies). Error bars represent ±SEM. E_max_ is reported with ±95% confidence intervals. mEnvy, monomeric Envy.

Above, we found that Ypk1 was important to internalization of Ste2 both in response to rapamycin and α-factor. Given the impact of Atg8 on rapamycin-induced receptor localization, we wondered whether Atg8 would also impact Ste2 during the pheromone response. Given that Atg8 promotes autophagosome formation in the cytosol (93), and autophagosomes ultimately fuse with the vacuole (94), we labeled the vacuole to assess endomembrane trafficking. We deleted *ATG8* from cells expressing Ste2-mEnvy and Vph1-Tomato and treated them with 10 μM pheromone for 2 h (Fig. 10*B*). The most notable effect we observed was that cells lacking Atg8 frequently had empty vacuoles. To quantify this, line scans were drawn through the center of the vacuole to measure the levels of Ste2-mEnvy at the vacuolar membrane, labeled by Vph1-Tomato, and the lumen (Fig. 10*A*). We found that deleting *ATG8* led to a 4-fold increase in cells with vacuoles lacking significant Ste2 accumulation (Fig. 10*C*). This would suggest that during mating, autophagy machinery contributes to the vacuolar targeting of Ste2.

Localization of Ste2 to the lumen of the vacuole would remove its cytoplasmic domain from the cytosol and stop any potential endomembrane signaling. We hypothesized that Atg8 and the autophagy machinery may therefore reduce pheromone signaling. To test this, we measured the pheromone dose–response curve with the pheromone-responsive FUS1 β-galactosidase assay described above to measure mating gene transcription in *atg8*Δ and WT cells (Fig. 10*D*). Cells lacking Atg8 showed a 30% increase in transcriptional output compared to WT cells, without shifting the EC_50._ Therefore, autophagy during the pheromone response suppresses the total pathway output of pheromone-induced transcription.

## Discussion

**Figure 11:**
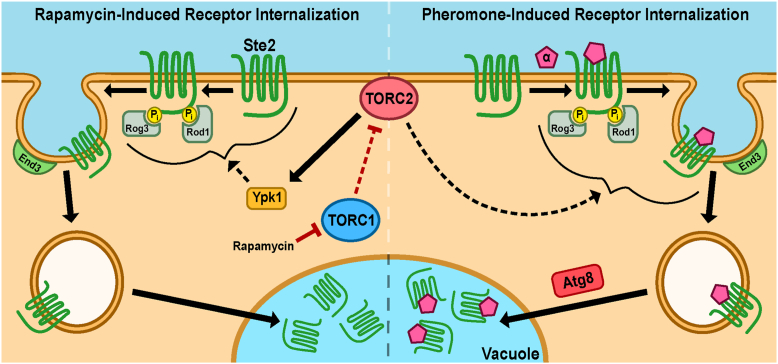
**Proposed mechanism of TORC1-directed endocytosis.** In nutrient-deprived conditions or during rapamycin-mediated inhibition, TORC1 is repressed, leading, through an unknown pathway, to the TORC2-directed activation of Ypk1. Active Ypk1 facilitates α-arrestin–directed CME of Ste2, which ultimately traffics to the vacuole. During the pheromone response, TORC2 is also required for endocytosis, after which Atg8 promotes trafficking of the receptor to the lumen of the vacuole. CME, clathrin-mediated endocytosis; mEnvy, monomeric Envy; TORC, target of rapamycin complex.

Here, we describe both ligand-dependent and ligand-independent regulation of the GPCR, Ste2, by TOR complexes and their effectors. We have shown that nutrient limitation leads to a decrease in the PM levels of mating pheromone receptors Ste2 and Ste3 in *S. cerevisiae*. We found that changes in TOR signaling are responsible for reduced Ste2 abundance on the PM, as rapamycin treatment phenocopies nitrogen limitation. Receptor internalization is concomitant with decreased signaling through the pheromone response pathway as well as a reduction in the ability of the yeast to mate. Through analysis of 15 different candidate genes by deletion analysis and additional truncations of Avo3 and Ste2 itself, we determined that the signaling pathway from TORC1 inhibition to loss of Ste2 from the PM was dependent upon TORC2-Ypk1 signaling and α-arrestin recruitment to the receptor, followed by End3-dependent CME (Fig. 11). Using pheromone-responsive transcriptional assays, we found that TORC1 signaling promotes pheromone signaling and that Ypk1 signaling suppresses it. Thus, TOR signaling regulates Ste2 PM abundance, mating pathway signaling, and mating itself. As TORC1 inhibition reduces mating, we propose that this signaling pathway serves as a mechanism to suppress mating during times of nutritional stress, when the metabolic requirements of mating may represent a risk to cell survival and fitness.

The link between TOR signaling, autophagy, and endomembrane transport led us to test whether autophagy machinery contributes to receptor trafficking to the vacuole during the pheromone response. We found that the central autophagy protein, Atg8, promotes vacuolar targeting of Ste2 and suppresses pheromone-dependent transcription. In the absence of nutritional stress, it is unexpected that autophagy machinery would play a role in the clearance of GPCRs from the cytosol. The finding that Atg8 promotes Ste2 trafficking to the vacuolar lumen and suppresses pathway output represents a novel mode of negative regulation of GPCR signaling.

### Why suppress mating with TOR?

Cells may reduce their mating receptor levels in response to starvation to avoid mating while hungry. There is an increased risk of cell death while responding to pheromone (53). If cells are stressed due to a lack of carbon sources and metabolites, mating may exacerbate this stress by committing significant energy and resources toward forming a mating projection. In this situation, it would be advantageous to prevent the mating response. While our quantitative mating assay showed only a 30% decrease in mating efficiency in response to rapamycin, this is a 5-h assay with a 5-fold excess of mating partners, a straightforward environment in which to mate. In single-cell assays, ∼50% of yeast can mate with an available partner within ∼30 min (98). As such, a 30% decrease over 5 h could mean a significant change in mating over the first 30 to 60 min, when most mating occurs.

Since Ste2 and Ste3 are at the start of the pathway, they are prime candidates for regulation. In this context, the observation that both Ste2 and Ste3 are removed from the PM suggests that the cell utilizes TOR signaling pathways to inhibit mating when nutrients are scarce, at least partially through receptor internalization. Ste2 has been shown to signal in a ratiometric manner, meaning that it is responsive to the fraction of occupied receptors rather than the absolute number of active receptors (99, 100). However, this requires receptor binding of the RGS, Sst2 (99). Sst2 does not colocalize with the receptor in the absence of pheromone and does not appear to accumulate prior to ∼30 min of pheromone treatment (82), giving a window for modulation of receptor levels to be more meaningful.

We found that Ypk1 was a robust negative regulator of the pheromone pathway (Fig. 8). We would not attribute this degree of regulation solely to its effect on receptor endocytosis, especially since Ypk1 deletion increased the Emax 4-fold, far more than deletion of other well-characterized negative regulators of the pathway (101). Therefore, we anticipate that Ypk1 is likely acting on the pheromone signaling pathway at other regulatory nodes as well to suppress the mating pathway.

### TOR-dependent endocytosis *versus* ligand-induced endocytosis

We have found that TORC1-induced endocytosis of Ste2 is a distinct process from pheromone-induced receptor internalization. During the pheromone response, Ste2 internalization is mediated primarily by three sets of proteins that interact with the receptor C terminus: 1) the yeast casein kinases Yck1 and Yck2 (57, 58, 59), 2) the α-arrestins Rog3 and Rod1 (56, 60, 61), and 3) the epsin-like proteins Ent1 and Ent2 (65, 66). Starvation-induced endocytosis aligns with receptor mediated internalization in that 1) the receptor C terminus is a required component, 2) it is mediated by α-arrestins, and 3) it is primarily clathrin-mediated as shown by our deletion screen hits (End3, Rog3, and Rod1) and Ste2^T326^ experiments. Cargo may be selected by α-arrestins to be internalized *via* either CME or clathrin-independent endocytosis (60). Thus, our arrestin data alone are insufficient to conclude that CME is used to internalize Ste2 during TORC1 inhibition. However, the impact of *END3* deletion indicates that Ste2 internalization is at least partly dependent upon CME. End3 is a major endocytic coat protein of clathrin pits (102) and its removal leads to a significant decrease in CME (83, 84). We showed that loss of End3 diminishes the rapamycin-induced Ste2 internalization significantly, but not entirely. This may be due to a drastically slowed, but still present CME, or alternatively a role for clathrin-independent endocytosis in receptor internalization during TORC1 inhibition.

Interestingly, the preferential use of arrestins in TORC1-mediated internalization makes this process unique from receptor-mediated endocytosis. Prior studies have shown that a double deletion of *ROG1* and *ROG3* cause significant defects in receptor-mediated Ste2 endocytosis, whereas single deletions of these genes show no significant effect, indicating that these proteins are redundant in their function during mating (56). However, we find that Rog3 is preferentially used in TORC1-mediated internalization. There are 33 serine and threonine residues in the C-terminal region of Ste2 that can be phosphorylated. Kim *et al.* found that specific residues contribute to different aspects of both receptor internalization and signaling during mating (87). Therefore, differential patterns of receptor phosphorylation may lead to preferential recruitment of endocytic adapter proteins, like Rog3, during starvation. Another key difference from ligand-induced internalization is that TORC1-mediated internalization does not seem to require Yck1 or Yck2. It is possible that this stems from the redundant functions of these kinases, with one kinase compensating for the other’s deficiency (57). However, another kinase, such as Ypk1, may be responsible for direct phosphorylation of the receptor during starvation. Regardless of the enzyme responsible for modifying the receptor, we have found differential use of arrestins that have previously been seen as redundant (56).

TORC2 is known to regulate endocytosis through multiple pathways (26, 30, 31, 34). The Hicke lab found that mutants in either Tor2 or Ypk1 result in reduced endocytosis of α-factor (30, 31). We find that TORC2 control of endocytosis must be shared between pheromone-induced and rapamycin-induced endocytosis of Ste2. This, unfortunately, complicates the elucidation of signals specific to the two stimuli. Regardless, this work provides additional evidence that TORC2 controls endocytosis of Ste2, expanding the context beyond pheromone-induced endocytosis to include TORC1-regulated endocytosis.

### Activation of TORC2 by inhibition of TORC1

We have found an interesting crossroad in TORC signaling, whereby TORC1 signaling appears to directly impact TORC2 activity. TORC1 and TORC2 are stress-responsive complexes that regulate cellular growth and metabolism. These complexes are structurally and functionally distinct. However, there are instances where TORC1 and TORC2 signaling pathways intertwine to tune the magnitude of their responses (103, 104). For example, while TORC1 is the primary regulator of autophagy during nitrogen starvation, TORC2 activity has been suggested to enhance autophagy specifically in the absence of amino acids (103). We have found that inhibition of TORC1 signaling drives Ste2 internalization that is dependent upon TORC2. This may represent a novel signaling pathway between TORC1 and TORC2 where one regulates the signaling of the other directly at the complex level, but further investigation of this interaction is required. We do not know if TORC1 directly affects TORC2, or, perhaps more likely, if the crosstalk is more circuitous.

Receptor internalization is a shared outcome to a variety of different stimuli, and in these different contexts, the shared components responsible may not be used in the same way. In this study, we have found that Ypk1 promotes receptor endocytosis when TORC1 activity is inhibited. Ypk1 is known to play a regulatory role in endocytosis through the TORC2 signaling pathway, but the nature of this regulation is not fully understood (30, 34, 105, 106). It has been reported that Ypk1 activity promotes receptor endocytosis in response to pheromone signaling (30), while others find that Ypk1 suppresses Rod1 interactions with Ste2, in a process predicted to repress Ste2 internalization (105). In this study, where the initiating stimulus is rapamycin inhibition of TORC1, Ypk1 promotes Ste2 internalization and does so through a Rog3-dependent process. Our studies provide yet another context where Ypk1 promotes endocytosis, but more work on Ypk1 and its control of endocytosis is required to determine how different signaling contexts impact their relationship.

Yeast initiates vacuolar targeting of cytoplasmic proteins during the pheromone response, which may represent engagement of selective autophagy such as cytoplasm-to-vacuole transport (74). One potential explanation is that, as a type of autophagy, it frees metabolites to support growth of the mating projection. However, our data suggest that it serves as a negative regulator of the signaling pathway. We have found that cells use Atg8 to dampen mating pathway output. Atg8 commonly associates with selective autophagy receptors (107, 108, 109, 110, 111). Perhaps mating stimulates autophagic clearance of cytosolic proteins to remove active receptor (and potentially other signaling molecules) from the cytosol. In mammalian cells, GPCR trafficking to various subcellular locations following internalization leads to unique signaling profiles (112, 113, 114, 115). It is possible that Ste2 may likewise signal at the endosome. In this scenario, the cell would rely upon Atg8 and autophagy to suppress endosomal signaling by directing Ste2 to the vacuole.

We found that TOR signaling impacted localization of both mating GPCRs in yeast. There are reports that β_2_-adrenergic receptor is targeted to the lysosome in a ligand-dependent manner in mammals (116). Together, these data suggest that TOR regulation of GPCRs may be broadly conserved. If so, drugs that target TOR in humans may impact the localization or signaling of at least a subset of human GPCRs. GPCRs and the regulation of their signaling is significant to human health and physiology, leading them to being the most targeted molecule by FDA-approved therapeutics (117). Therefore, understanding signaling cascades that control their output is crucial to therapeutic development for diseases that may arise from dysregulated GPCR signaling, such as heart failure and certain cancers (118, 119, 120). These studies open the doors to avenues of research at the intersection of GPCR and TOR signaling.

## Experimental procedures

### Yeast strains

Strains reported in this study are listed in Table S1. All strains were constructed in the MATa haploid *S. cerevisiae* parent strain BY4741, except for those used to visualize Ste3. In which case, strains were constructed in the MATα haploid parent strain BY4742. Strains containing fluorescent labeled, deleted, or truncated genes were created through oligonucleotide-directed homologous recombination, using primers listed in Table S2. Primer amplicons were generated through PCR using Q5 Hot Start High-Fidelity 2X Master Mix (M0494, NEB). GPCRs (Ste2, Ste3, Gpr1) and Ypk1 were labeled with an mEnvy (mEnvy/Envy^V206K^) (121). EGFP sourced from pFA6a-link-yoEGFP-SpHis5 (Addgene Plasmid #44836) (122) was used to label and truncate Ste2 simultaneously. Pma1 labeling is discussed below. Genetic deletion mutants and the Avo3 truncation strains were generated by replacing the target locus with a kanamycin resistance cassette (KanMX6) that can be selected for *via* geneticin treatment. This cassette was sourced from a Yeast Deletion Library (Dharmacon) or from the pFA6a-yo-linkEGFP-KanMX plasmid (Addgene Plasmid #44900) (122).

Cells were grown in yeast extract peptone dextrose (YPD) growth media at 30 °C. PCR products were transformed into parent yeast strains using a standard lithium acetate transformation with single-stranded carrier DNA and PEG (123). These cultures were grown on standard selective media (SCD his-, SCD leu-, SCD ura-, or YPD G418+) and isolated *via* auxotrophic or geneticin resistance-based selection. Transformants were verified using PCR and fluorescence microscopy when applicable. PCR verification was performed using One*Taq* 2X Master Mix with Standard Buffer (M0482, NEB) or LongAmp Hot Start *Taq* 2X Master Mix (M0533, NEB).

### Yeast cell culture for microscopy

Cells were grown overnight in 0.22 μm-filtered SCD media at 30 °C to an A_600_ of 0.2 to 0.8. To limit cells of nitrogen sources, cells were diluted from SCD media into low nitrogen SCD media (SCD ammonium sulfate-, uracil-, L-histidine-, L-leucine-) and incubated for 4.5 h prior to imaging. To repress TORC1 activity with rapamycin (0215934691, MP Biomedicals), cells were treated with 0.2 μM rapamycin suspended in ethanol for 2 h prior to imaging. To induce the mating response, cells were treated with 10 μM α-factor (RP01002, GenScript) suspended in autoclaved milliQ water for 2 h prior to imaging.

### Live-cell widefield microscopy

All cells were imaged on 2% agarose pads in SCD. Agarose pads were supplemented with the same concentrations of rapamycin or α-factor as in liquid cultures where applicable. Cells were imaged using an inverted widefield fluorescence microscope (IX83, Olympus) equipped with a Prime 95B CMOS camera (Teledyne Photometrics). All images were acquired using a UPlanXApo 100X/1.45NA Oil objective (N5702400, Olympus). Z-stack images were collected with a step size of 0.32 μm.

Transmitted light illumination was performed using a 12 V 100 W halogen light bulb at 5.0 V power. Transmitted light exposure time was 200 ms. Fluorescence illumination was performed using an X-Cite 120 LEDBoost (Excelitas) at 40% light intensity. EGFP and mEnvy emission was collected using Ex466/40 Em525/50 filter set (GFP-4050B-OFF-ZERO, Semrock) at 3 s exposure.

Images were acquired at 200X magnification with an IX3 magnification changer, using the Z before channel setting, with image size being 1200 × 1200 pixels. The software was cellSens Dimension 2.3 (Olympus, https://evidentscientific.com/en/products/software/cellsens), and images were saved as 16 bit VSI files.

### Analysis of peripheral GPCR abundance

Z-stacks of fluorescent and differential interference contrast (DIC) images were acquired as described above. In FIJI, a single slice of the fluorescent and DIC images in the middle of the Z-stack were isolated and saved as 16 bit TIFF files to be used for mask generation and analysis. These images were merged and qualitatively assessed as quality control to ensure that the isolated fluorescent and DIC frame exist in the same focal plane. The saved DIC images were loaded into Cellpose 2.0 (124) for mask generation. In Cellpose 2.0, the cyto2 pretrained model was used to generate masks for individual cells in each image, with a user-defined cell diameter of 80 pixels, a flow threshold (flow_threshold) of 0, and a mask threshold (cellprob_threshold) of 1.5. Any masks that did not encompass the entire cell were fixed or removed. Any masks that overlaid debris, dead cells, or cells that were partially out of frame were removed. Masks were saved as PNG files.

Masks were loaded into MATLAB and eroded with a diamond structuring element (1-pixel radius) to ensure individual cell masks are not touching. Masks were converted into a logical mask, and all connected components with fewer than 5000 pixels were removed. The logical mask was then converted back to a “double” data format. To convert these into masks that encompass the cell periphery, the masks were first duplicated. The duplicate masks were eroded with a diamond structuring element (7-pixel radius), and the new area encompassed by these masks were removed from the originals. This would leave behind a working mask which covers the edge of each cell. Converting this to a count mask generates the working mask used to measure the fluorescent intensity along the cell edge in individual cells.

Single-slice fluorescent images were loaded into MATLAB. A Gaussian Blur was applied to the image with a standard deviation (Sigma) of 50 and subtracted from the original image (performing a pseudo-flat field background subtraction). The mean fluorescent intensity was calculated from this image within the area defined by the working mask described above for each individual cell.

### Statistical analysis of epifluorescence microscopy data

In experiments where two sample groups were being compared to each other (Figs. 1*D* and 2*E*), two-tailed two-sample *t* tests of unequal variance were performed. Any *p* value produced that was less than 0.05 deemed the two groups to have a difference in means that is statistically significant. In experiments where greater than two groups were being compared (Figure 3, Figure 4, Figure 5, Figure 6, Figure 7*B* and 9*B*), one-way ANOVAs were performed. Each ANOVA compared four groups: WT untreated cells, WT cell treated with rapamycin, mutant untreated cells, and mutants treated with rapamycin. The ANOVA results were then processed through a multiple comparison test (Tukey’s honestly significant difference test). As described above, a *p* value equal to or less than 0.05 denoted a significant difference between sample groups.

### Mating gene transcription assays

Assays were performed based on the β--galactosidase Reporter Gene Assay (Liquid Form) from the Dohlman Lab (80). Cells were transformed with the plasmid pRS423-pFUS1-LacZ as previously described. Colonies were isolated by growth on standard selective media (SCD his-). Cultures were grown in SCD his-media at 30 °C to an A_600_ of 0.1 to 0.8. Cells were pelleted at 2500 rpm for 5 min and resuspended in SCD his-media to obtain an A_600_ of 0.6 to 0.8. In a polystyrene 96-well plate, 90 μl of cells were added to each well per row. Each row of wells containing cells represented an individual biological replicate, as these cells were grown from different transformant colonies. Then, 10 μl of α-factor was added to each well, whereby each column of wells contained different concentrations of the α-factor: for example, 0 μM, 0.003 μM, 0.01 μM, 0.03 μM, 0.1 μM… 300 μM. Cells were gently shaken and incubated at 30 °C for 90 min. Solutions of 1 mM fluorescein di-β-galactopyranoside (F1179, Invitrogen) diluted in 25 mM potassium phosphate buffer pH 7.2 and 0.5% Triton X-100 diluted in 250 mM potassium phosphate buffer pH 7.2 were mixed in equal parts. After the 90 min incubation, 20 μl of this solution was added to each well; cells were gently shaken, covered in aluminum foil, and incubated at 37 °C for 90 min. The reaction was quenched with 20 μl of 1 M sodium bicarbonate and read on a fluorescent plate reader (BioTek Synergy 2, Agilent) at an excitation wavelength of 485/20 and emission wavelength of 528/20. Fluorescent intensity values were normalized to cell culture density and averages were fit to a Hill Equation using the Curve Fitting Toolbox 3.8 in MATLAB to determine the E_max_, EC_50_, and Hill slope of the generated dose response curve with their respective 95% confidence intervals. Differences in these values were considered statistically significant if their confidence intervals did not overlap. Lower bounds of these values were set to zero and upper bounds remained infinite.

### Quantitative mating assays

Assays were performed based on the quantitative mating assays described by Sprague Jr *et al.* (81). Cultures of MATa (BY4741) and MATα (BY4742) cells were grown in YPD at 30 °C overnight to an A_600_ of 0.2 to 0.8. Cell densities of these cultures in cells/ml were then determined using a hemocytometer. Then, 10^7^ MATα cells were mixed with 2 × 10^6^ MATa cells. This suspension was then filtered through a 0.45-μm pore 25-mm diameter nitrocellulose filter disk, such that the liquid media was discarded, leaving the cells to lie on the filter. The filter was then transferred to an YPD plate (YPD rapamycin + if cells were pretreated with 0.2 μM rapamycin) and incubated at 30 °C for 5 h. Cells were then resuspended in 2 ml of SCD media and further diluted such that a countable number of colonies was achieved once plated. Cells were plated on YPD and incubated at 30 °C until colonies were visible (1 day for untreated cells, 2 days for rapamycin-treated cells). Cells were then replica plated onto SCD lys- and SCD lys-met- cys- to select for MATa + diploid cells and diploid cells, respectively. These plates were incubated 30 °C for 1 day, and colonies from each plate were counted. Mating efficiency was determined by calculating the percentage of cells that mated (SCD lys-met-cys- colonies) out of all mating competent cells in the mating suspension (SCD lys- colonies). To determine whether the mean mating efficiencies of rapamycin-treated and untreated groups were significantly different, we performed a one-tailed two-sample *t* test of unequal variance. A *p* value of less than 0.05 was considered statistically significant.

### Live-cell confocal microscopy

All cells were imaged on 2% agarose pads in SCD. Agarose pads were supplemented with 10 μM α-factor, the same concentration as in liquid cultures. Images were acquired using a point scanning confocal unit (LSM 980, Carl Zeiss Microscopy) on a Zeiss Axio Observer 7 inverted microscope (409000-9663-000, Carl Zeiss Microscopy) equipped with a C Plan-Apochromat 63X/1.4NA Oil objective lens (421782-9900-799, Carl Zeiss Microscopy). Samples were placed into an Incubator XLmulti S2 DARK (ref: 411857-9310-000, Carl Zeiss Microscopy) and warmed at 30 °C using the Temp Module S1 (ref: 411860-9010-000, Carl Zeiss Microscopy) and the heating unit XL S2 (ref: 411857-9031-000, Carl Zeiss Microscopy). Z-stack images were collected with a step size of 0.17 μm with the Motorized Scanning Stage 130 × 100 STEP Set LSM (ref: 409000-9420-000, Carl Zeiss Microscopy) mounted on the universal mounting frame K-M (ref: 432341-9100-000, Carl Zeiss Microscopy) and the insert mounting frame K-M for specimen slides (ref: 432340-9040-000, Carl Zeiss Microscopy).

mEnvy fluorescence was excited with a 488 nm diode laser at 3% power and collected using an Airyscan 2 detector at 800 V with 495 to 555 nm BP and 660 nm long pass filters through a pinhole at 5.25 AU. ytdTomato fluorescence was excited with a 561 nm diode laser at 3% power and collected using an Airyscan 2 detector at 850 V with 420 to 480 nm band pass and 570 to 630 band pass filters through a pinhole at 5.00 AU. Airyscan images were processed manually in 3D with a strength of the deconvolution set to 7.5.

Images were frame-scanned sequentially in SR mode, with a pixel time of 0.66 μs, a frame time of 486.6 μs, a zoom factor of 4X, using bidirectional scanning. Images were acquired such that each track was acquired before moving to the next Z-step. Images were 768 × 768 pixels, each pixel being 0.043 × 0.043 μm. The software was Zen Blue 3.8 (Carl Zeiss Microscopy, https://www.zeiss.com/microscopy/us/products/software/zeiss-zen.html), and images were saved as 16 bit CZI files.

### Analysis of GPCR abundance at the vacuolar membrane and lumen

Using FIJI, background was subtracted from GFP and RFP images with a rolling ball radius of 50 pixels. Line scans were drawn through the largest vacuolar body in each cell. The mean fluorescent intensities of Vph1-Tomato (vacuolar membrane) and Ste2-mEnvy (mating receptor) were measured at each position across the line scans. Using MATLAB, the two local maxima of Vph1-Tomato fluorescence and their immediately flanking positions were labeled as the vacuolar membrane. At these positions, the mean fluorescent intensities of Ste2-mEnvy were averaged to determine receptor levels at the vacuolar membrane. Between these positions, Ste2-mEnvy intensities were averaged to determine receptor levels in the vacuolar lumen. For each cell, Ste2 levels at the vacuolar membrane were divided by receptor levels in the lumen. Cells were then classified as expressing phenotype 1 (full vacuoles) if their lumen:membrane ratios were greater than 1. Cells were classified as expressing phenotype 2 (empty vacuoles) if their lumen:membrane ratios were less than 1. Violin plots were generated in MATLAB using violin plot-MATLAB (125).

### Mutagenesis of GFP Envy in an epitope-tagging plasmid

The pFA6a-link-GFPEnvy-SpHis5 epitope-tagging plasmid (Addgene Plasmid #60782) (121) was mutagenized such that the expressed Envy protein was incapable of dimerization. Val206 of Envy was mutagenized to Lys with the Q5 Site-Directed Mutagenesis Kit (E0554S, NEB) using primers listed in Table S2. The mutated product was transformed into chemically competent *Escherichia coli* through a High Efficiency Transformation (C2987H, NEB). Colonies were isolated *via* growth on selective media (LB carbenicillin+). Colonies were grown in liquid selective media, and plasmids were purified using a Monarch Plasmid Miniprep Kit (T1010, NEB). Successful mutagenesis was verified through DNA sequencing (Plasmidsaurus).

### Cloning pRSII405-*PMA1-mRUBY2*

A plasmid construct containing *PMA1-mRUBY2* was generated in a pRSII405 vector *via* Gibson Assembly. The sequence for *PMA1* was amplified from BY4741 genomic DNA, and the *mRUBY2* sequence was amplified from pFA6a-link-yomRuby2-Kan (Addgene Plasmid #44953) (122). Both amplicons were generated through PCR with Q5 Hot Start High-Fidelity 2X Master Mix (M0494, NEB) using primers listed in Table S2. The vector, pRSII415 (Addgene Plasmid #35440) (126), was digested by SacII (R1057, NEB) and HindIII-HF (R3104, NEB). The cut vector was verified *via* band separation using gel electrophoresis, and the vector was extracted from the gel using a Monarch DNA Gel Extraction Kit (T1020, NEB). Gibson Assembly of the three generated fragments was performed using NEBuilder HiFi DNA Assembly Master Mix (E2621, NEB). The resulting assembly mixture was transformed into chemically competent *E. coli* through a High Efficiency Transformation (C2987H, NEB). Colonies were isolated *via* growth on selective media (LB carbenicillin+). Colonies were grown in liquid selective media, and plasmids were purified using a Monarch Plasmid Miniprep Kit (T1010, NEB). Constructs were then verified by restriction digest using SacI-HF (R3156, NEB) and SalI-HF (R3138, NEB) and through DNA sequencing (Plasmidsaurus). Verified plasmids were digested BamHI-HF (R3136, NEB) and transformed into yeast using the transformation methods described above. Colonies were grown on standard selective media (SCD leu-) and isolated *via* auxotrophic selection. Transformants were verified using PCR and fluorescence microscopy. PCR verification was performed using One*Taq* 2X Master Mix with Standard Buffer (M0482, NEB).

## Data availability

MATLAB scripts used for the analysis of receptor localization at the plasma membrane are available on GitHub (https://github.com/Kelley-Lab-Computational-Biology).

## Supporting information

This article contains supporting information.

## Conflict of interest

The authors declare that they have no conflicts of interest with the contents of this article.
