# Supplementary material for: TOR signaling regulates GPCR levels on the plasma membrane and suppresses the Saccharomyces cerevisiae mating pathway
Source: J Biol Chem. 2025 Sep 11;301(10):110700. doi: 10.1016/j.jbc.2025.110700 (PMC12547020; doi:10.1016/j.jbc.2025.110700)
Supplement: Supporting Tables and Figures [file mmc1.pdf]

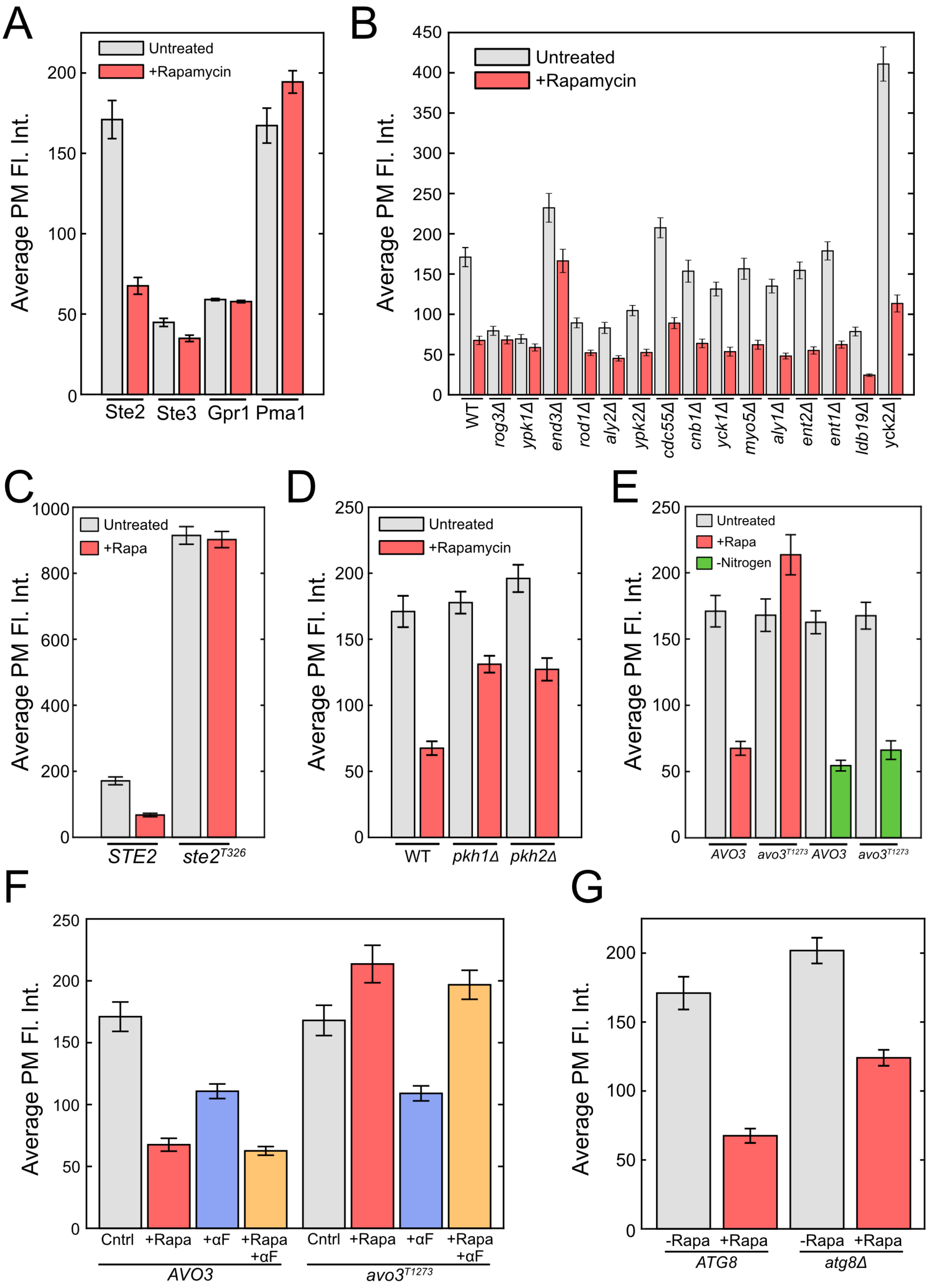
A
Untreated
+Rapamycin
Average PM Fl. Int.
Ste2
Ste3
Gpr1
Pma1
B
Untreated
+Rapamycin
Average PM Fl. Int.
WT
rog3Δ
ypk1Δ
end3Δ
rod1Δ
aly2Δ
ypk2Δ
cdc55Δ
cnb1Δ
yck1Δ
myo5Δ
aly1Δ
ent2Δ
ent1Δ
ldb19Δ
yck2Δ
C
Untreated
+Rapa
Average PM Fl. Int.
STE2
ste2T326
D
Untreated
+Rapamycin
Average PM Fl. Int.
WT
pkh1Δ
pkh2Δ
E
Untreated
+Rapa
-Nitrogen
Average PM Fl. Int.
AVO3
avo3T1273
AVO3
avo3T1273
F
Average PM Fl. Int.
Cntrl
+Rapa
+αF
+Rapa +αF
AVO3
avo3T1273
G
Average PM Fl. Int.
-Rapa
+Rapa
ATG8
atg8Δ

Figure S1. Plasma membrane intensity data without normalization. (**A**) Non-normalized data reported in Figure 2. (**B**) Non-normalized data reported in Figure 3. (**C**) Non-normalized data reported in Figure 4. (**D**) Non-normalized data reported in Figure 5. (**E**) Non-normalized data reported in Figure 6. (**F**) Non-normalized data reported in Figure 7. (**G**) Non-normalized data reported in Figure 9. All error bars represent S.E.M.

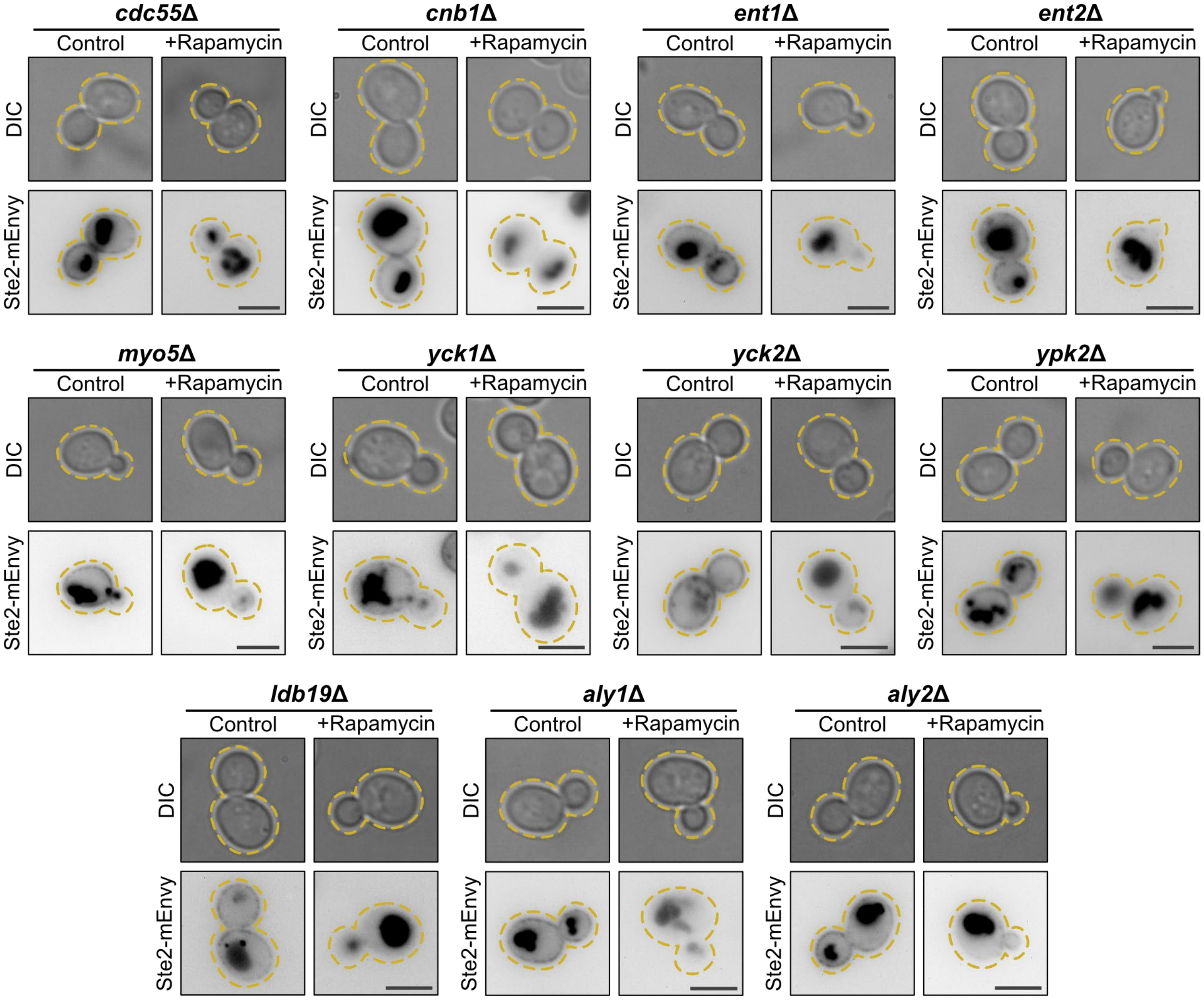

Figure S2. Single genetic deletions that do not block rapamycin-induced endocytosis. Representative images of genetic deletion mutants that do not block Ste2 internalization in the presence of 0.2µM rapamycin. Scale bars represent 4µm.

Table S1. Strain List.

Strain	***Parent***	***Description***
BY4741	S288C	*his3Δ1*<br>*leu2Δ0*<br>*met15Δ0*<br>*ura3Δ0*
BY4742	S288C	*his3Δ1*<br>*leu2Δ0*<br>*lys2Δ0*<br>*ura3Δ0*
STE2-mEnvy	BY4741	*STE2:Envy*^{V206K}*::HIS3*
STE3-mEnvy	BY4742	*STE3:Envy*^{V206K}*::HIS3*
GPR1-mEnvy	BY4741	*GPR1:Envy*^{V206K}*::HIS3*
PMA1-mRuby2	BY4741	*PMA1:mRuby2::LEU2*
STE2-mEnvy aly1Δ	BY4741	*STE2:Envy*^{V206K}*::HIS3*<br>*aly1Δ::KanMX6*
STE2-mEnvy aly2Δ	BY4741	*STE2:Envy*^{V206K}*::HIS3*<br>*aly2Δ::KanMX6*
STE2-mEnvy cdc55Δ	BY4741	*STE2:Envy*^{V206K}*::HIS3*<br>*cdc55Δ::KanMX6*
STE2-mEnvy cnb1Δ	BY4741	*STE2:Envy*^{V206K}*::HIS3*<br>*cnb1Δ::KanMX6*
STE2-mEnvy end3Δ	BY4741	*STE2:Envy*^{V206K}*::HIS3*<br>*end3Δ::KanMX6*
STE2-mEnvy ent1Δ	BY4741	*STE2:Envy*^{V206K}*::HIS3*<br>*ent1Δ::KanMX6*
STE2-mEnvy ent2Δ	BY4741	*STE2:Envy*^{V206K}*::HIS3*<br>*ent2Δ::KanMX6*
STE2-mEnvy ldb19Δ	BY4741	*STE2:Envy*^{V206K}*::HIS3*<br>*ldb19Δ::KanMX6*
STE2-mEnvy myo5Δ	BY4741	*STE2:Envy*^{V206K}*::HIS3*<br>*myo5Δ::KanMX6*

STE2-mEnvy rod1Δ	BY4741	*STE2:Envy^{V206K}::HIS3 rod1Δ::KanMX6*
STE2-mEnvy rog3Δ	BY4741	*STE2:Envy^{V206K}::HIS3 rog3Δ::KanMX6*
STE2-mEnvy yck1Δ	BY4741	*STE2:Envy^{V206K}::HIS3 yck1Δ::KanMX6*
STE2-mEnvy yck2Δ	BY4741	*STE2:Envy^{V206K}::HIS3 yck2Δ::KanMX6*
STE2-mEnvy ypk1Δ	BY4741	*STE3:Envy^{V206K}::HIS3 ypk1Δ::KanMX6*
STE2-mEnvy ypk2Δ	BY4741	*STE2:Envy^{V206K}::HIS3 ypk2Δ::KanMX6*
ste2^{T326}-EGFP	BY4741	*ste2^{T326}:EGFP::HIS3*
ypk1Δ	BY4741	*ypk1Δ::KanMX6*
STE2-mEnvy avo3^{T1273}	BY4741	*STE2:Envy^{V206K}::HIS3 avo3^{T1273}::KanMX6*
STE2-mEnvy pkh1Δ	BY4741	*STE2:Envy^{V206K}::HIS3 pkh1Δ::KanMX6*
STE2-mEnvy pkh2Δ	BY4741	*STE2:Envy^{V206K}::HIS3 pkh2Δ::KanMX6*
STE2-mEnvy VPH1-Tomato	BY4741	*STE2:Envy^{V206K}::HIS3 VPH1:ytdTomato::URA3*
STE2-mEnvy VPH1-Tomato atg8Δ	BY4741	*STE2:Envy^{V206K}::HIS3 VPH1:ytdTomato::URA3 atg8Δ::KanMX6*
STE2-mEnvy atg8Δ	BY4741	*STE2:Envy^{V206K}::HIS3 atg8Δ::KanMX6*
atg8Δ	BY4741	*atg8Δ::KanMX6*

Table S2. Primer List.

Primer Name	*Sequence*	*Description*
WSM-7	5' GGAAGCCAGAAAGTTCTGGACTGAAGATAATAATAATTTAGGTGACGGTGCTGGTTTA 3'	Amplify fluorescent label to tag Ste2
WSM-8	5' GAAGGTCACGAAATTACTTTTTCAAAGCCGTAAATTTTGATCGATGAATTCGAGCTCG 3'	Amplify fluorescent label to tag Ste2
WSM-11	5' GATGCTAAAAGCAGTCTCAG 3'	Verify Ste2 labeling
WSM-12	5' GAGAGTTCTAGATCATGGCA 3'	Verify Ste2 labeling
NLM-1	5' CGAGGTCGACGGTATCGATACCAAAGGCTAAGGACGCTTTG 3'	Amplify *PMA1* ORF for Gibson Assembly
NLM-2	5' CACCGTCACCGGTTTCCTTTTCGTGTTGAGTAG 3'	Amplify *PMA1* ORF for Gibson Assembly
NLM-3	5' AAAGGAAACCGGTGACGGTGCTGGTTTAATTAAC 3'	Amplify *mRUBY2* ORF for Gibson Assembly
NLM-4	5' AAAAGCTGGAGCTCCACCGCTTACTTATACAATTCATCCATACCACC 3'	Amplify *mRUBY2* ORF for Gibson Assembly
NLM-5	5' AAGCTCAAACGAACATAGTTCAGAAAATACTGCAGGCCCTGGTGACGGTGCTGGTTTA 3'	Amplify fluorescent label to tag Ste3
NLM-6	5' AATACTCCTAGTCCAGTAAATATAATGCGACACTCTTGTGTCGATGAATTCGAGCTCG 3'	Amplify fluorescent label to tag Ste3
NLM-7	5' CGGCATAGATTTGATAGCCTTCTTAAGAAATGGACCATTAGGTGACGGTGCTGGTTTA 3'	Amplify fluorescent label to tag Gpr1
NLM-8	5' TTCCTTACTTTCCATTTTCAAACATCGCGATACAAAAACTTCGATGAATTCGAGCTCG 3'	Amplify fluorescent label to tag Gpr1
NLM-9	5' ACACCAATATCACAAGCGCA 3'	Verify Ste3 labeling
NLM-10	5' TCTGCTAATCGACTTTTGGAGC 3'	Verify Ste3 labeling
NLM-11	5' TCTACCCGGGTTGAAATTTGC 3'	Verify Gpr1 labeling

NLM-12	5’ ACGAGCACTCATCCATTTTCA 3’	Verify Gpr1 labeling
NLM-15	5’ AGAACCGACCAAGACGTGTT 3’	Amplify deletion cassette at *ATG8* ORF from Yeast Deletion Library
NLM-16	5’ GTACGTTAAGAACAGCGGCA 3’	Amplify deletion cassette at *ATG8* ORF from Yeast Deletion Library
NLM-35	5’ TCCCTCGTTCACAGAAAGTCT 3’	Verify Pma1 labeling
NLM-36	5’ TGCATCACAGGTCCGTTAGA 3’	Verify Pma1 labeling
NLM-63	5’ CCTTTTGATGTTACCCCGCC 3’	Amplify deletion cassette at *YCK1* ORF from Yeast Deletion Library
NLM-64	5’ AAAGGGGCAAAGGTGTGAAG 3’	Amplify deletion cassette at *YCK1* ORF from Yeast Deletion Library
NLM-65	5’ TCCACGTAGAACATCGCAGT 3’	Verify *YCK1* deletion
NLM-66	5’ ACAGGAATCGAATGCAACCG 3’	Verify *YCK1* deletion
NLM-67	5’ TCATTAAAGTGTGGGCTGTGG 3’	Amplify deletion cassette at *YCK2* ORF from Yeast Deletion Library
NLM-68	5’ TGCAAATTGAAAGAGGGTAAACA 3’	Amplify deletion cassette at *YCK2* ORF from Yeast Deletion Library
NLM-69	5’ GTCTTGATGCTCTGAAGGCG 3’	Verify *YCK2* deletion
NLM-70	5’ TCCGACTCGTCCAACATCAA 3’	Verify *YCK2* deletion
NLM-71	5’ ACCAACAGTCCGCACATAGA 3’	Verify *YPK1* deletion

NLM-72	5’ CCATGAGACACAAGCCACAC 3’	Verify *YPK1* deletion
NLM-79	5’ AGTTCCTGTTTTGCCAATGGT 3’	Amplify deletion cassette at *MYO5* ORF from Yeast Deletion Library
NLM-80	5’ GGAATTACCGACGCTCCATT 3’	Amplify deletion cassette at *MYO5* ORF from Yeast Deletion Library
NLM-81	5’ TTGAGTTCTGCCGTTCAAGC 3’	Verify *MYO5* deletion
NLM-82	5’ TAACGGCTCCAAGATTGTGC 3’	Verify *MYO5* deletion
NLM-83	5’ TGGGTCAAATTATCGCGTATACAAATATACATA TAGTAACGACATGGAGGCCCAGAATAC 3’	Amplify deletion cassette with homology to *YPK2* UTRs
NLM-84	5’ AAATTCCGTCCGGCTCGGCTCGGCTTGCTTCG GCTTGCTTCAGTATAGCGACCAGCATTC 3’	Amplify deletion cassette with homology to *YPK2* UTRs
NLM-85	5’ TGGCGTGGTTGAACATCTTG 3’	Verify *YPK2* deletion
NLM-86	5’ TCCGACTCGTCCAACATCAA 3’	Verify *YPK2* deletion (with NLM-85), *ROD1* deletion (with RAM-15), *ROG3* deletion (with RAM-17), and *ENT1* deletion (with SLM-7)
NLM-89	5’ CCGATGAGGCAAAATATGGTGT 3’	Verify *ATG8* deletion
NLM-90	5’ CGAACTCTTTCCCATTGTCTGT 3’	Verify *ATG8* deletion
NLM-91	5’ GAGGGGTCAGAAGATGCAGA 3’	Amplify deletion cassette at *ALY1* ORF from Yeast Deletion Library
NLM-92	5’ CCGGTACTTTTCCCAGACGA 3’	Amplify deletion cassette at *ALY1* ORF from Yeast Deletion Library

NLM-93	5’ ATTCTGGGGAGGAGCAAGTC 3’	Verify *ALY1* deletion
NLM-94	5’ CCAACCCGAGGAGAAATTGC 3’	Verify *ALY1* deletion
NLM-95	5’ ACGCCTTCACCTATCACTCT 3’	Amplify deletion cassette at *ALY2* ORF from Yeast Deletion Library
NLM-96	5’ GCGGGAAGAAGTCAAAAGACA 3’	Amplify deletion cassette at *ALY2* ORF from Yeast Deletion Library
NLM-97	5’ GATGTCGAACGGAGAGCAAC 3’	Verify *ALY2* deletion
NLM-98	5’ TTGGAACGGCTGAAGAAACG 3’	Verify *ALY2* deletion
NLM-99	5’ CACATCAAGACCACTGCGAG 3’	Amplify deletion cassette at *CNB1* ORF from Yeast Deletion Library
NLM-100	5’ AGATGGTCTGTCTCCTAGCA 3’	Amplify deletion cassette at *CNB1* ORF from Yeast Deletion Library
NLM-101	5’ GGGAAATGGGTTGTGGACTT 3’	Verify *CNB1* deletion
NLM-102	5’ AATCAGCGGGTTTCCTCCTT 3’	Verify *CNB1* deletion
NLM-107	5’ AGCTCCACCTCAAAGACCAA 3’	Verify *CDC55* deletion
NLM-108	5’ TCCGACTCGTCCAACATCAA 3’	Verify *CDC55* deletion
NLM-111	5’ GGAGAGATCTTACGCATAAAGAAATATAATATAG CGCACAGACATGGAGGCCCAGAATAC 3’	Amplify deletion cassette with homology to *CDC55* UTRs
NLM-112	5’ GGGATAAAAAAAAGTAAGGGAAAATAAGGAATT ATTATAACAGTATAGCGACCAGCATTC 3’	Amplify deletion cassette with homology to *CDC55* UTRs

NLM-115	5’ GTTCCTCTCATATCAACAAACATTAATACAGTTCCTGAAAGACATGGAGGCCCAGAATAC 3’	Amplify deletion cassette with homology to *YPK1* UTRs
NLM-116	5’ TATGTCATGAGTAAACTAGTTGATAATGTATTCACTAAGTCAGTATAGCGACCAGCATTC 3’	Amplify deletion cassette with homology to *YPK1* UTRs
NLM-117	5’ TACAATTTCTTTGTATACAGCGGGAAATTGACACTTCAAAGTTCCTCTCATATCAACAAA 3’	Extend homology of NLM-115/NLM-116 amplicon to *YPK1* UTR.
NLM-118	5’ AGTAAGTAACGGAAAAGAAAACCTTTCTTTTTATATAAAGTATGTCATGAGTAAACTAGT 3’	Extend homology of NLM-115/NLM-116 amplicon to *YPK1* UTR.
NLM-150	5’ AACAGAAGGGTGCCAACATG 3’	Verify *PKH1* deletion
NLM-151	5’ CCTGAAATACATGCCCGAAGA 3’	Verify *PKH1* deletion
NLM-154	5’ GCGAACATTTCCCGATCCTT 3’	Amplify deletion cassette at *PKH2* ORF from Yeast Deletion Library
NLM-155	5’ AAGCGTTGCCTTTGTGAGC 3’	Amplify deletion cassette at *PKH2* ORF from Yeast Deletion Library
NLM-156	5’ AGCTATTGACGAAGGCCCAT 3’	Verify *PKH2* deletion
NLM-157	5’ GCAGTTAATCCAAGGTGCCA 3’	Verify *PKH2* deletion
NLM-162	5’ AGCGGATGGTCGTTCTTCTC 3’	Verify *END3* deletion
NLM-163	5’ GCTGAAGCGTGAGAATGAGT 3’	Verify *END3* deletion
NLM-166	5’ AAGCGATGGCGACTTGATAGAAAAATGCTTACCCATCTAGGACATGGAGGCCCAGAATAC 3’	Amplify deletion cassette to truncate *AVO3*
NLM-167	5’ TTGTGACTATATACATTTATACATGCGGCCCTTTTTTGCTCAGTATAGCGACCAGCATTC 3’	Amplify deletion cassette to truncate *AVO3*

NLM-168	5' AGCCTTAGAAACCGACACGA 3'	Verify *AVO3* truncation
NLM-169	5' TCTGCTGAAACGGAACTCCC 3'	Verify *AVO3* truncation
NLM-187	5' GCACGTGTACTTGCTTGAATACTGCTACTA TATCATTAATGACATGGAGGCCCAGAATAC 3'	Amplify deletion cassette with homology to *PKH1* UTRs
NLM-188	5' TGTCTTACATATGCATATATATATATTATCAA GCACAGTTCAGTATAGCGACCAGCATTC 3'	Amplify deletion cassette with homology to *PKH1* UTRs
NLM-189	5' TATTGGAAAGGCCGGTAAAGATAACAGGGATCTC TGAAAAGACATGGAGGCCCAGAATAC 3'	Amplify deletion cassette with homology to *END3* UTRs
NLM-190	5' AAATATTACACATTCATGTACATAAAATTAATTATC GGTGCAGTATAGCGACCAGCATTC 3'	Amplify deletion cassette with homology to *END3* UTRs
NLM-193	5' TATAATCAGACGATTGTAATATAACCATAGAGTTAG TGGGTATTGGAAAGGCCGGTAAAG 3'	Extend homology of NLM-189/NLM-190 amplicon to *END3* UTR.
NLM-194	5' TTCAAATCAAAAAAGTTTACAAGTGAAATAACAAA CAGTAAATATTACACATTCATGTAC 3'	Extend homology of NLM-189/NLM-190 amplicon to *END3* UTR.
NLM-242	5' GGAAGTCGCTGTTGCTAGTGCAAGCTCT TCCGCTTCAAGCGGTGACGGTGCTGGTTTA 3'	Amplify fluorescent label to tag Vph1
NLM-243	5' AGTACTTAAATGTTTCGCTTTTTTTAAAAG TCCTCAAAATTCGATGAATTCGAGCTCG 3'	Amplify fluorescent label to tag Vph1
NLM-244	5' CCCTCATCAAGCAAAGGTCC 3'	Verify Vph1 labeling
NLM-245	5' GAACACCATCAACGGATCGA 3'	Verify Vph1 labeling
RAM-1	5' CCCTACCCGACTGCAAACTA 3'	Amplify deletion cassette at *ROD1* ORF from Yeast Deletion Library
RAM-2	5' TGTTGAGGAAGAAGTGCCAAG 3'	Amplify deletion cassette at *ROD1* ORF

		from Yeast Deletion Library
RAM-5	5’ GTTATGGACCCGGAGAGAGG 3’	Amplify deletion cassette at *ROG3* ORF from Yeast Deletion Library
RAM-6	5’ GTGTCGCAGTCCATAGAAGG 3’	Amplify deletion cassette at *ROG3* ORF from Yeast Deletion Library
RAM-9	5’ GGCCAGGTTGCTAGTGACTA 3’	Amplify deletion cassette at *LDB19* ORF from Yeast Deletion Library
RAM-10	5’ TGCCCACCCTTTATTTTGCC 3’	Amplify deletion cassette at *LDB19* ORF from Yeast Deletion Library
RAM-13	5’ ACCGTTGATGCTGATGAGGA 3’	Verify *LDB19* deletion
RAM-14	5’ CCCTCAAGCATCGCAGTTT 3’	Verify *LDB19* deletion
RAM-15	5’ CAAATCACTACAAGCCCGCA 3’	Verify *ROD1* deletion (with NLM-86)
RAM-17	5’ AGTATCGATGCGCTGAGTGA 3’	Verify *ROG3* deletion (with NLM-86)
SLM-3	5' TAGGCTTCTTACCGGCAGAG 3'	Amplify deletion cassette at *ENT1* ORF from Yeast Deletion Library
SLM-4	5' AGGACATGAGTACAGAGCACA 3'	Amplify deletion cassette at *ENT1* ORF from Yeast Deletion Library
SLM-5	5' AGATAGATGGCCGAACGTGG 3'	Amplify deletion cassette at *ENT2* ORF from Yeast Deletion Library
SLM-6	5' GACTCCAAAGGTGAATCTGGC3'	Amplify deletion cassette at *ENT2* ORF from Yeast Deletion Library

SLM-7	5' GGGAGAAGGAGTACCTTCTG 3'	Verify *ENT1* deletion
SLM-10	5' CGAAGATGGCCGAATGATTGG 3'	Verify *ENT2* deletion
SLM-21	5’ TGTGGGCATCTCTGGTTTGA 3’	Verify *ENT2* deletion
CJM-1	5’ ACAACATCCACAGATAGGTTTTATCCAGGC ACGCTGTCTAGCGGTGACGGTGCTGGTTTA 3’	Amplify fluorescent label to tag and truncate Ste2
CJM-2	5’ CGAAGGTCACGAAATTACTTTTTCAAAGC CGTAAATTTTGACGATGAATTCGAGCTCGTT 3’	Amplify fluorescent label to tag and truncate Ste2
CJM-3	5’ CAATGTGGGCCACGGCTGCTAA 3’	Verify labeling and truncation of Ste2
CJM-4	5’ ACAGCGTACCTTTAGACACGTGGG 3’	Verify labeling and truncation of Ste2
JKM-70	5’ AACCCAATCTaagCTATCTAAAGACC 3’	Mutate Envy Val^{206} to Lys
JKM-71	5’ GATAGGTAGTGGTTGTCTG 3’	Mutate Envy Val^{206} to Lys

Table S3. Statistical Results of Figure 7.

Comparison Group #1	*Comparison Group #2*	*p-Value*
AVO3 Control	*AVO3* +Rapamycin	8.52E-14
AVO3 Control	*AVO3* +Pheromone	0.001138838
AVO3 Control	*AVO3* +Rapamycin +Pheromone	5.70E-16
AVO3 Control	*avo3*^{T1273} Control	1
AVO3 Control	*avo3*^{T1273} +Rapamycin	0.016305491
AVO3 Control	*avo3*^{T1273} +Pheromone	0.002441116
AVO3 Control	*avo3*^{T1273} +Rapamycin +Pheromone	0.358141005
AVO3 +Rapamycin	*AVO3* +Pheromone	0.04131578
AVO3 +Rapamycin	*AVO3* +Rapamycin +Pheromone	0.999910346
AVO3 +Rapamycin	*avo3*^{T1273} Control	1.67E-10
AVO3 +Rapamycin	*avo3*^{T1273} +Rapamycin	0
AVO3 +Rapamycin	*avo3*^{T1273} +Pheromone	0.047081906
AVO3 +Rapamycin	*avo3*^{T1273} +Rapamycin +Pheromone	0
AVO3 +Pheromone	*AVO3* +Rapamycin +Pheromone	0.010376711
AVO3 +Pheromone	*avo3*^{T1273} Control	0.00592741
AVO3 +Pheromone	*avo3*^{T1273} +Rapamycin	2.62E-12
AVO3 +Pheromone	*avo3*^{T1273} +Pheromone	0.99999994
AVO3 +Pheromone	*avo3*^{T1273} +Rapamycin +Pheromone	5.86E-09
AVO3 +Rapamycin +Pheromone	*avo3*^{T1273} Control	5.70E-12
AVO3 +Rapamycin +Pheromone	*avo3*^{T1273} +Rapamycin	0
AVO3 +Rapamycin +Pheromone	*avo3*^{T1273} +Pheromone	0.01287587
AVO3 +Rapamycin +Pheromone	*avo3*^{T1273} +Rapamycin +Pheromone	0
avo3^{T1273} Control	*avo3*^{T1273} +Rapamycin	0.054865881
avo3^{T1273} Control	*avo3*^{T1273} +Pheromone	0.010098816
avo3^{T1273} Control	*avo3*^{T1273} +Rapamycin +Pheromone	0.53058797
avo3^{T1273} +Rapamycin	*avo3*^{T1273} +Pheromone	2.72E-11
avo3^{T1273} +Rapamycin	*avo3*^{T1273} +Rapamycin +Pheromone	0.895798206
avo3^{T1273} +Pheromone	*avo3*^{T1273} +Rapamycin +Pheromone	3.47E-08
